# Oral antibiotic exposure and urinary tract infection risk in adults: a self-controlled case series study

**DOI:** 10.1016/j.eclinm.2026.104060

**Published:** 2026-07-14

**Authors:** Mirjam Deelen, Willian J. van Dijk, Mirte Boelens, Hanneke Borgdorff, Merel MC. Lambregts, Cees van Nieuwkoop, Martijn Sijbom

**Affiliations:** a Department of Public Health and Primary Care, Leiden University Medical Center, Health Campus The Hague, The Hague, the Netherlands; b Department of Internal Medicine, Haga Teaching Hospital, The Hague, the Netherlands

**Keywords:** Urinary tract infection, Infectious diseases, Antibiotic exposure, Microbiome, Self-controlled case series study

## Abstract

**Background:**

Antibiotic treatment, central to bacterial infection management, may increase urinary tract infection (UTI) risk by disrupting the microbiome. This study assessed whether oral antibiotic treatment, regardless of indication, is associated with increased UTI incidence in adults.

**Methods:**

A self-controlled case series study using routine-care data (2015–2024) from the Extramural LUMC Academic Network, comprising >140 general practices in the Netherlands. Adults (≥18 years) with ≥1 recorded UTI were included; individuals with <1 year of registration were excluded. Exposure was defined as an oral antibiotic treatment. UTIs were identified using International Classification of Primary Care version 1 (ICPC-1) codes for cystitis (U70) and acute pyelonephritis (U71), or UTI symptoms recorded ≤5 days of antibiotic treatment. Distinct antibiotic treatments were ≥14 days apart or had different Anatomical Therapeutic Chemical (ATC) codes. Conditional Poisson regression analyses, stratified by sex, age, and antibiotic group, estimated incidence rate ratios (IRRs) by comparing UTI incidence during risk periods (15–30, 15–90, 15–180, and 15–365 days after antibiotics) with the control period (365-15 days before antibiotics).

**Findings:**

Among 82,337 adults, 206,428 UTIs were recorded, mainly in women (79.9%) and older adults (54.8% in women >50 and 58.4% in men >65 years). The IRR was highest within 30 days (IRR: 1.74; 95% CI: 1.73–1.76): 1.98 in women ≤50 years (95% CI: 1.93–2.02), 1.52 in women >50 years (95% CI: 1.50–1.54), 3.47 in men ≤65 years (95% CI: 3.31–3.63), 2.42 in men >65 years (95% CI: 2.36–2.50), and 1.96 for β-lactams (95% CI: 1.91–2.01).

**Interpretation:**

Oral antibiotic treatment was associated with a modest increase in UTI incidence in both sexes, with the highest risk observed within 30 days. These findings may be considered when prescribing antibiotics in primary care, particularly in otherwise healthy individuals.

**Funding:**

LUMC funded the study without study role.

**Research in context**

**Evidence before this study**

Urinary tract infections (UTIs) are common bacterial infections. Established risk factors include e.g., sex and age. Additionally, antibiotic exposure has been suggested to increase subsequent UTI risk, potentially through microbiome disruption, particularly in women, whose vaginal microbiota contributes to colonisation resistance. Previous cohort studies, primarily in women <50 years old, reported varying estimates of increased UTI risk following antibiotic exposure, ranging from approximately 1.1-fold to up to six-fold. However, no large, population-based studies have examined UTI risk following antibiotic exposure while accounting for factors such as sex, age, antibiotic group, and distinct risk periods.

**Added value of this study**

This large, population-based self-controlled case series study extends previous evidence by assessing the association between oral antibiotic treatment and subsequent UTI incidence across a broader population, including both women and men. Using routine-care data from over 200,000 UTIs, it provides more generalizable estimates than prior studies conducted in selected populations. The findings indicate a modest association between antibiotic treatment and UTI incidence (IRR: 1.03; 95% CI: 1.03–1.04), particularly within 30 days (IRR: 1.74; 95% CI: 1.73–1.76), and reveal variation by sex, age, and antibiotic group (e.g., IRR within 30 days, in men ≤65 years old, and β-lactams: 3.14; 95% CI: 2.86–3.44).

**Implications of all the available evidence**

Taken together, existing evidence and the present findings suggest a modest association between oral antibiotic treatment and subsequent UTI incidence, with a relatively higher UIT risk observed within 30 days. Although the overall increase in UTI risk is small, the high frequency of antibiotic prescribing in primary care may have population-level relevance. Since the hypothesized mechanism of microbiome disruption may not be specific to UTIs, these findings may be considered as an additional factor in antibiotic prescribing decisions in primary care, particularly in otherwise healthy individuals.

## Introduction

Urinary tract infections (UTIs) are common bacterial infections, with an estimated 404 million UTIs reported worldwide in 2019.^1^ In the Netherlands, UTI incidence in 2023 was 117 per 1000 women and 20 per 1000 men.^2^ Factors associated with UTI risk, besides sex,^3^^,^^4^ include age (>50 years old),^4^ previous UTIs,^3^ urinary tract abnormalities,^3^^,^^4^ and sexual activity.^3^ In women, prior antibiotic exposure has also been suggested to increase UTI risk.^5^ In 2022, Dutch general practitioners (GPs) prescribed a median of 245 antibiotic treatments per 1000 patients,^6^ most commonly β-lactams and largely for respiratory tract infections (RTIs).^7^^,^^8^ Despite low antimicrobial resistance (AMR) in the Netherlands, resistance to commonly prescribed antibiotics persists, underscoring the importance of selective antibiotic prescribing.^7^^,^^8^ Together, the burden of UTIs, AMR, and the possibility that antibiotic exposure may increase infection risk highlight the need to clarify the association between antibiotic treatment and UTI incidence.

Antibiotic exposure may disrupt the microbiome, the diverse microbial communities (i.e., microbiota) that support host defences against infection,^5^ thereby increasing UTI risk.^9^, ^10^, ^11^, ^12^ Broad-spectrum antibiotics, such as β-lactams, which target many bacterial species, may disrupt the microbiome and increase resistance gene abundance (i.e., the resistome), with effects lasting weeks to months.^13^, ^14^, ^15^, ^16^ Even narrow-spectrum antibiotics, which target fewer bacterial species, may disrupt the microbiome, albeit to a lesser extent.^13^, ^14^, ^15^, ^16^, ^17^ These disruptions may be particularly relevant in women, whose vaginal microbiota contributes to defences against UTIs.^5^ Dominated by lactobacilli, the vaginal microbiota maintains an acidic environment and produces antimicrobial compounds that prevent pathogen colonisation, providing colonisation resistance.^18^ Antibiotic exposure may reduce lactobacilli abundance, weakening these defences.^5^

Although biologically plausible, population-based studies investigating the association between antibiotic treatment and UTI incidence remain limited and have focused exclusively on women.^9^, ^10^, ^11^, ^12^ Smith et al. (1999) conducted a prospective cohort study among 781 women aged 18–50 years old and observed that antibiotic exposure in the 15–28 days before a UTI was associated with a three-to six-fold increase in UTI risk.^9^ Margolis et al. (2005) performed a retrospective cohort study among 118,496 individuals aged 15–35 years old receiving long-term antibiotic treatment for acne and observed that, in women, antibiotic treatment was associated with 1.1 times higher odds of UTI.^10^ Similarly, Cai et al. (2012) performed a prospective cohort study among 673 women aged 18–40 years old receiving antibiotic treatment for asymptomatic bacteriuria (ASB) and observed that antibiotic exposure was associated with a one-to three-fold increase in UTI risk.^11^ Rich et al. (2019) conducted a retrospective cohort study among 6620 women aged 18–25 years old with UTIs and observed that antibiotic exposure was associated with 1.0–2.1 times higher odds of recurrence.^12^

Collectively, these studies suggest that antibiotic treatment may increase UTI incidence in women, but large, population-based studies assessing this association across sex, age, and antibiotic groups are lacking. The present study addresses this gap by assessing whether oral antibiotic treatment is associated with an increased UTI incidence in adults in the year following antibiotic exposure compared with the year prior, and whether this association varies by sex, age, or antibiotic group.

## Methods

### Study design

**Fig. 1:**
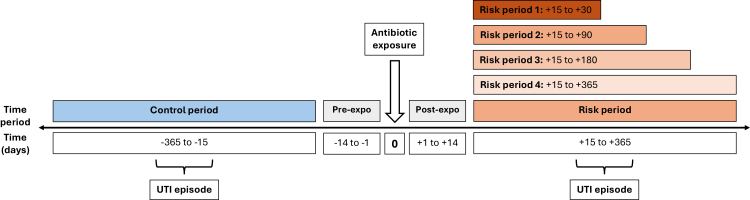
**Schematic representation of the self-controlled case series study design.** Illustrating one antibiotic treatment (exposure) and two urinary tract infections (UTI; outcome). Control period: 365–15 days before antibiotic exposure (blue). Risk period: 15–365 days after antibiotic exposure (orange), divided into risk period 1 (15–30 days), risk period 2 (15–90 days), risk period 3 (15–180 days), and risk period 4 (15–365 days). Pre-exposure period: 14–1 days before antibiotic exposure (grey). Post-exposure period: 1–14 days after antibiotic exposure (grey).

A self-controlled case series (SCCS) design was used to compare, within individuals, UTI risk across predefined time periods (Fig. 1).^19^^,^^20^ For each antibiotic exposure, a 730-day observation period was defined, spanning 365 days before to 365 days after the prescription date. The control period comprised the 365–15 days before exposure. The pre-exposure period comprised the 14–1 days before exposure; UTIs during this period were excluded to minimize event-dependent exposure bias. The post-exposure period comprised the 1–14 days after exposure; UTIs during this period were excluded to minimize reverse causation bias. The risk period comprised the 15–365 days after exposure, divided into risk period 1 (15–30 days), risk period 2 (15–90 days), risk period 3 (15–180 days), and risk period 4 (15–365 days). Risk period 4 represented the complete risk period following antibiotic exposure, mirroring the duration of the control period. Each antibiotic exposure defined a separate observation period and was analysed individually. Individuals with multiple antibiotic exposures could contribute multiple observation periods (Supplementary Material Fig. 1).

A cohort was assembled of individuals aged ≥18 years old with at least one UTI between January 1, 2015 and December 31, 2024. Two washout periods (January 1, 2015–January 1, 2016 and December 31, 2023–December 31, 2024) were applied to minimize effects of prior antibiotic treatments and ensure complete observation periods near study boundaries. All UTIs recorded between the washout periods were included to allow all eligible UTIs to contribute to the within-person comparisons and to reduce bias when outcomes are common or baseline risk varies across subgroups.^19^^,^^21^ Follow-up spanned 365 days before the first antibiotic exposure to 365 days after the last, or until loss to follow-up, death, or study end. Individuals were excluded if sex was missing or unknown, if they were <18 years old at the antibiotic prescription date, or if they had less than one year of GP practice registration before their first antibiotic exposure. Analyses were restricted to oral antibiotic treatments, the most commonly prescribed form in primary care.^7^^,^^8^

### Data source & cohort

Pseudonymized routine-care data were obtained from the Extramural LUMC Academic Network (ELAN), a collaboration between healthcare providers and the Department of Public Health and Primary Care of the Leiden University Medical Center (LUMC).^22^ ELAN contains electronic health records for approximately one million patients from >140 GP practices in the Leiden and the Hague regions, the Netherlands.^22^ This study was determined not to be subject to the Dutch Medical Research Involving Human Subjects Act and was approved by the Medical Ethics Committee of the LUMC (reference nr. 25–3005). The study protocol was additionally approved by the Scientific Review Board of the Department of Public Health and Primary Care of the LUMC. Patients were informed through GP materials (e.g., leaflets) and could opt out, after which their data were excluded from ELAN. This study complied with the Dutch Civil Code (Article 7:458) and the General Data Protection Regulation (Article 9.2.j).^22^

### Variables

Antibiotic treatments were identified from the ELAN medication dataset using the World Health Organisation Anatomical Therapeutic Chemical (ATC) codes, with all oral antibiotics (J01) grouped into a single variable.^23^ Antibiotic treatments were subsequently classified as either exposures or outcomes. Treatments prescribed for indications other than UTIs, or, when the indication was missing, not accompanied by a recorded UTI symptom within five days, were classified as exposures. Treatments prescribed for UTI diagnoses, or, when the indication was missing, accompanied by a recorded UTI symptom within five days, were considered to represent UTIs and were subsequently classified as outcomes. Antibiotic treatments were considered distinct if ATC codes differed or, if identical, were prescribed ≥14 days apart by prescription date.

UTIs were identified from the ELAN episodes dataset using International Classification of Primary Care version 1 (ICPC-1) codes.^24^ UTI diagnoses, including acute pyelonephritis (U70) and cystitis (U71), and UTI-related symptoms were grouped into a single variable. UTI-related symptoms, including dysuria (U01), urinary frequency/urgency (U02), other micturition problems (U05), other urinary symptoms/complaints (U07), other bladder symptoms/complaints (U13), and abnormal urine test result (U98), were included only if recorded within five days of an antibiotic treatment with a missing indication. UTIs were considered distinct if ICPC-1 codes differed or, if identical, were recorded ≥14 days apart by registration date.

Potential effect modifiers included sex (women and men),^3^^,^^4^ age at the antibiotic prescription date (≤50 and >50 years old for women and ≤65 and >65 years old for men),^4^ and antibiotic group (all J01 subgroups).^5^^,^^13^, ^14^, ^15^, ^16^, ^17^

### Statistical analyses

Analyses were performed in RStudio (version 4.3.1). Cohort characteristics were summarised using medians with interquartile ranges (IQRs) or counts with percentages. Person-time contributed to each time period was calculated in years. Conditional Poisson regression with a logarithmic link function was used to estimate associations between oral antibiotic treatment and UTI incidence, in which an offset term was included to account for varying observation time across distinct risk periods. Incidence rates (IRs) were described for the purpose of providing insight into how IRRs were calculated. The study protocol and statistical analysis plan are available in the Supplementary Material: Research Protocol.

### Primary analyses

In the primary analyses, including all antibiotic treatments per individual, the UTI incidence during each distinct risk period was compared with the UTI incidence during the control period (Fig. 1). Effect modification was assessed through stratified analyses based on variables selected a priori, informed by the literature and biological plausibility. Stratified analyses were performed by sex, age, antibiotic group, and combined sex-, age-, and antibiotic group categories. Combined sex- and age categories included women ≤50 (i.e., premenopausal) and >50 years old (i.e., postmenopausal), a proxy for menopausal status and the associated increase in UTI risk, as well as men ≤65 and >65 years old, reflecting a comparable increase in UTI risk. Antibiotic groups included β-lactams, macrolides/lincosamides/streptogramins (hereafter: macrolides), other antibacterials (i.e., ATC-code J01X “other antibacterials”), quinolones, sulphonamides/trimethoprim (hereafter: sulphonamides), and tetracyclines. Subgroups with at least 500 UTIs were retained for subgroup analyses.

### Sensitivity analyses

Four sensitivity analyses were performed.^22^ Time-adjusted 1 analyses extended the pre-and post-exposure periods to 30 days to address the assumption that outcomes should not influence the exposure (Fig. 1). Time-adjusted 2 analyses shortened the observation period to 360 days (spanning 180 days before to 180 days after antibiotic exposure) to address the assumption that outcome risk remained stable within the time periods (Fig. 1). Censoring analyses excluded observation periods in which individuals deregistered from their GP practice or died ≤365 days after antibiotic exposure. Random antibiotic analyses included only one (sole or randomly selected) antibiotic exposure per individual, to assess the influence of overlapping observation periods (Supplementary Material Fig. 1).

### Funding

This study was funded by the Department of Public Health and Primary Care of the LUMC, the Netherlands. The funder had no role in the design or conduct of the study, or the decision to submit the manuscript for publication.

## Results

**Fig. 2:**
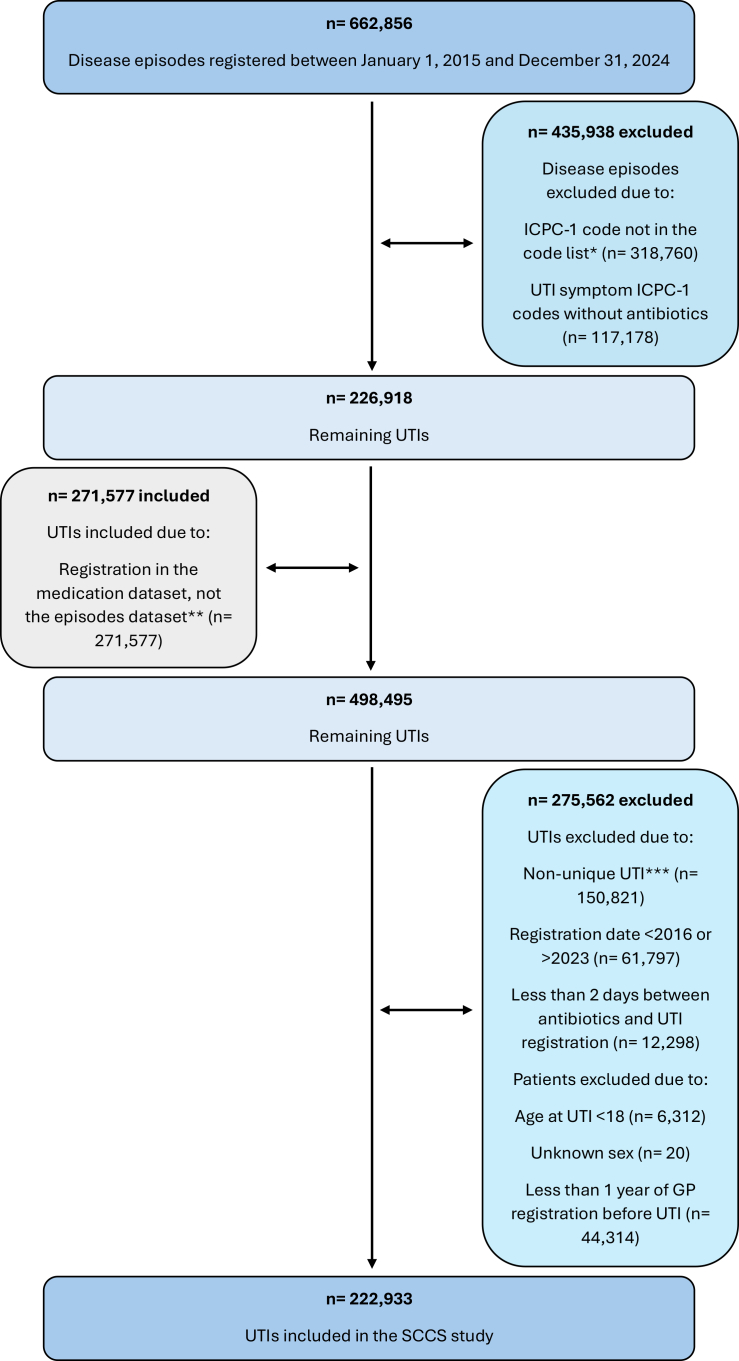
**Flow diagram of the cohort assembly.** Illustrating the assembly of urinary tract infections (UTI) for the self-controlled case series (SCCS) study. ∗ICPC-1 codes excluded for not being in the exposure definition code list (see *Methods: Variables*). For a full overview of these ICPC-1 codes, see Supplementary Material Table 1. ∗∗UTIs were deemed unique if they had different ICPC-1 codes or, if identical, were ≥14 days apart by registration date (see *Methods: Variables*).

A total of 662,856 disease episodes were recorded in the ELAN episodes dataset between January 1, 2015 and December 31, 2024. After applying inclusion criteria, 222,933 UTIs were identified (Fig. 2; Supplementary Material Table 1). Of these UTIs, 206,428 occurred within the 730-day observation period spanning an antibiotic exposure and were included in the analysis. The included UTIs were contributed by 82,337 unique individuals, with a median of 2 UTIs per individual (IQR: 1–3) (Table 1). Most UTIs occurred in women (79.9%), of whom 54.8% were postmenopausal. Among men, 58.4% of UTIs occurred in those >65 years old. In total, 439,816 antibiotic treatments were prescribed, corresponding to a median of 3 antibiotic treatments per individual (IQR: 2–5) (Table 1).

**Table 1:** Characteristics of individuals, urinary tract infections, antibiotic treatments, and person-time in the self-controlled case series study.

Characteristic	n (%) or median (IQR)
Study population	
Unique individuals	82,337
Women	65,786 (79.9%)
≤50 years old	29,756/65,786 (45.2%)
>50 years old	36,030/65,786 (54.8%)
Men	16,551 (20.1%)
≤65 years old	6891/16,551 (41.6%)
>65 years old	9660/16,551 (58.4%)
Urinary tract infections	
Unique urinary tract infections	206,428
Urinary tract infections per individual	2 (1–3)
Antibiotic treatments	
Unique antibiotic treatments	439,816
Antibiotic treatments per individual	3 (2–5)
Person-time (years)^a^	
Control period (365–15 days)^b^	422,656.85
Complete risk (15–365 days)^c^	422,656.85
Risk period 1 (15–30 days)	19,266.41/422,656.85 (4.6%)
Risk period 2 (15–90 days)	91,515.44/422,656.85 (20.7%)
Risk period 3 (15–180 days)	199,889.00/422,656.85 (47.3%)
Risk period 4 (15–365 days)	422,656.85/422,656.85 (100.0%)
General practitioner consults^d^	
Consults per individual in 2018	7 (4–13)
Consults per individual in 2024	8 (4–15)

IQR, indicates interquartile range.

a Person-time represents the total time (in years) contributed to each time period.

b Control period: spanning 365–15 days before antibiotic exposure.

c Complete risk period: spanning 15–365 days after antibiotic exposure, divided into: risk period 1 (15–30 days), risk period 2 (15–90 days), risk period 3 (15–180 days), and risk period 4 (15–365 days) following antibiotic exposure.

d General practitioner consults include physical, telephone, email, and e-consults.

Person-time was equal in the control and complete risk periods, comprising 442,656.85 person-years each (Table 1). Overall, UTI incidence increased from 1.08 per person-year in the control period to 1.12 per person-year in the risk period (Supplementary Material Table 2). Among women, UTI incidence increased from 0.72 to 0.83 per person-year in premenopausal women and remained stable in postmenopausal women (IR: 1.32 and 1.31, respectively). Among men, UTI incidence increased from 0.63 to 0.78 per person-year in those ≤65 and from 0.91 to 0.96 per person-year in those >65 years old (Supplementary Material Table 3). Across antibiotic groups, one of the largest increases in UTI incidence was observed for β-lactams, from 0.92 to 1.00 per person-year (Supplementary Material Table 4).

### Primary analyses

**Fig. 3:**
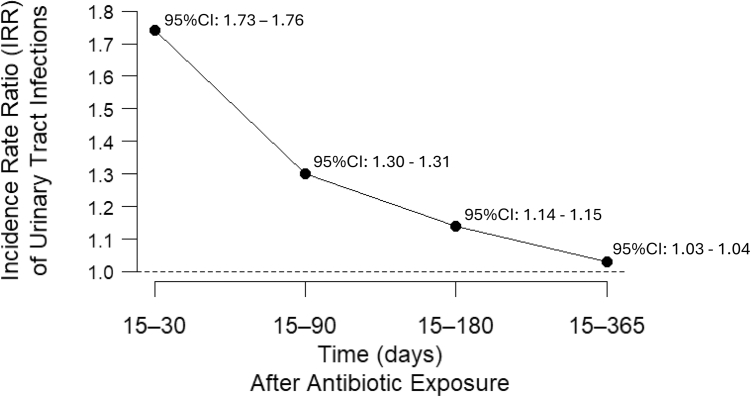
**Incidence rate ratio plot of the primary analysis.** Incidence rate ratio (IRR) plot of the primary analysis, including all antibiotic exposures per individual, of the self-controlled case series study assessing the association between oral antibiotic treatment and urinary tract infection incidence. Divided into risk period 1 (15–30 days), risk period 2 (15–90 days), risk period 3 (15–180 days), and risk period 4 (15–365 days) following antibiotic exposure.

In the primary analyses, the IRR was 1.74 (95% CI: 1.73–1.76) in risk period 1, 1.30 (95% CI: 1.30–1.31) in risk period 2, 1.15 (95% CI: 1.14–1.15) in risk period 3, and 1.03 (95% CI: 1.03–1.04) in risk period 4 (Fig. 3).

**Fig. 4:**
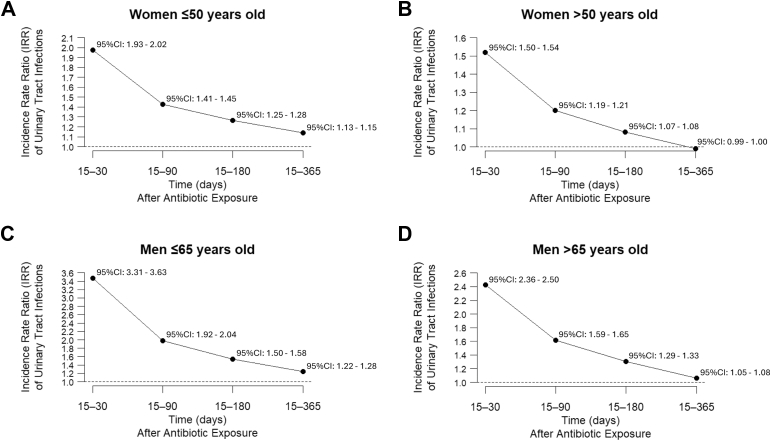
**Incidence rate ratio plot of the sex-and age-stratified primary analysis.** A: primary analysis stratified by women ≤50 years old. B: primary analysis stratified by women >50 years old. C: primary analysis stratified by men ≤65 years old. D: primary analysis stratified by men >65 years old. Incidence rate ratio (IRR) plot of the primary analysis, including all antibiotic exposures per individual, of the self-controlled case series study assessing the association between oral antibiotic treatment and urinary tract infection incidence. Divided into risk period 1 (15–30 days), risk period 2 (15–90 days), risk period 3 (15–180 days), and risk period 4 (15–365 days) following antibiotic exposure and stratified by sex and age: women ≤50, women >50, men ≤65, and men >65 years old.

In the sex- and age-stratified analyses, the IRR was highest during risk period 1 across all subgroups. Among women, the IRR was 1.98 (95% CI: 1.93–2.02) for premenopausal women and 1.52 (95% CI: 1.50–1.54) for postmenopausal women. Among men, the IRR was 3.47 (95% CI: 3.31–3.63) for those ≤65 and 2.42 (95% CI: 2.36–2.50) for those >65 years old (Fig. 4).

In the antibiotic group stratified analyses, the IRR was highest during risk period 1 for all antibiotic groups; the IRR for β-lactams was 1.96 (95% CI: 1.91–2.01) (Supplementary Material Table 4).

**Fig. 5:**
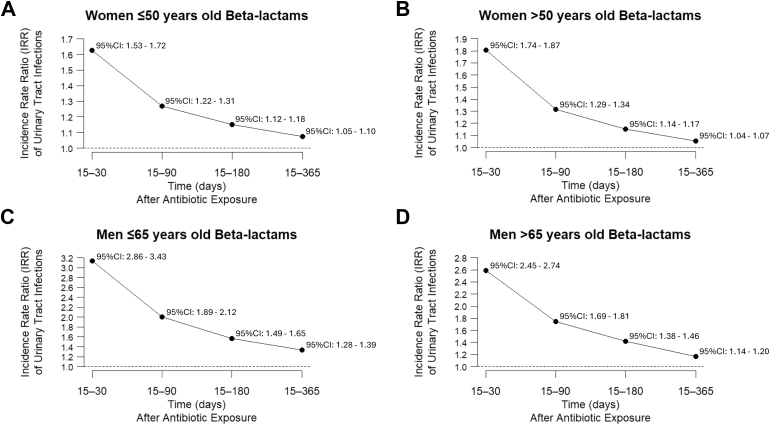
**Incidence rate ratio plot of sex-, age-, and β-lactam-stratified primary analysis.** A: primary analysis stratified by women ≤50 years old and β-lactams. B: primary analysis stratified by women >50 years old and β-lactams. C: primary analysis stratified by men ≤65 years old and β-lactams. D: primary analysis stratified by men >65 years old and β-lactams. Incidence rate ratio (IRR) plot of the primary analysis, including all antibiotic exposures per individual, of the self-controlled case series study assessing the association between oral antibiotic treatment and urinary tract infection incidence. Divided into risk period 1 (15–30 days), risk period 2 (15–90 days), risk period 3 (15–180 days), and risk period 4 (15–365 days) following antibiotic exposure, stratified by sex and age: women ≤50, women >50, men ≤65, and men >65 years old and antibiotic group β-lactams.

Results from the sex-, age-, and antibiotic group stratified analyses are presented for β-lactams. The IRR was highest during risk period 1 across all subgroups: 1.63 (95% CI: 1.53–1.72) for premenopausal women, 1.81 (95% CI: 1.74–1.87) for postmenopausal women, 3.14 (95% CI: 2.86–3.43) for men ≤65 years old, and 2.59 (95% CI: 2.45–2.74) for men >65 years old (Fig. 5; Supplementary Material Table 5).

### Sensitivity analyses

In the time-adjusted 1 and time-adjusted 2 analyses, results were consistent with the primary analyses (Supplementary Material Tables 6–13). Censoring occurred in 0.26% of observation periods, and the censoring analyses results were consistent with the primary analyses (Supplementary Material Tables 14–17).

Across all observation periods, the median overlap was 2 (IQR: 1–4) overlapping observation periods per individual. In the random antibiotic analyses, the IRR was 3.34 (95% CI: 3.23–3.45) in risk period 1, 1.92 (95% CI: 1.88–1.97) in risk period 2, 1.49 (95% CI: 1.46–1.52) in risk period 3, and 1.23 (95% CI: 1.21–1.25) in risk period 4 (Supplementary Material Table 18).

In the sex- and age-stratified analyses, the IRR was highest during risk period 1 across all subgroups. Among women, the IRR was 2.96 (95% CI: 2.77–3.17) for premenopausal women and 2.80 (95% CI: 2.67–2.94) for postmenopausal women. Among men, the IRR was 8.15 (95% CI: 7.31–9.10) for those ≤65 and 4.90 (95% CI: 4.50–5.32) for those >65 years old (Supplementary Material Table 19).

In the antibiotic group stratified analyses, the IRR was highest during risk period 1 for nearly all antibiotic groups. Among β-lactams, the IRR was 2.70 (95% CI: 2.50–2.92) (Supplementary Material Table 20).

Results from the sex-, age-, and antibiotic group stratified analyses are presented for β-lactams. The IRR was highest during risk period 1 across all subgroups: 1.60 (95% CI: 1.34–1.91) for premenopausal women, 2.64 (95% CI: 2.35–2.97) for postmenopausal women, 5.25 (95% CI: 4.20–6.57) for men ≤65 years old, and 4.00 (95% CI: 3.39–4.72) for men >65 years old (Supplementary Material Table 21).

## Discussion

The present study assessed whether oral antibiotic treatment is associated with increased UTI incidence in adults. A modest association was observed, with the highest UTI risk within 30 days after antibiotic exposure, particularly in men ≤65 years old and for antibiotic groups such as β-lactams. Although higher UTI risk was observed in men, women, particularly those >50 years old, continued to experience the highest UTI incidence.

Few prior studies have investigated the association between antibiotic treatment and UTI incidence. Smith et al. (1999) reported a stronger association, potentially reflecting diary-based data capturing mild or unverified UTIs less consistently recorded in routine-care data.^9^ Margolis et al. (2005) observed a smaller association, possibly due to continuous antibiotic exposure obscuring temporal variation in UTI risk.^10^ Cai et al. (2012) and Rich et al. (2019) reported associations of similar magnitude to the present findings.^11^^,^^12^ The present study extends previous evidence by including stratified analyses by sex, age, and antibiotic group. UTI risk was assessed across distinct risk periods using a large, population-based sample and within-person comparisons, providing a more detailed investigation of UTI incidence following antibiotic treatment.

The observed associations appear consistent with evidence that antibiotic treatment may disrupt the microbiome, potentially increasing susceptibility to infection.^9^, ^10^, ^11^, ^12^ Microbiome disruption may weaken colonisation resistance for weeks to months, potentially enabling pathogen overgrowth.^13^, ^14^, ^15^, ^16^, ^17^^,^^25^ The modest UTI risk observed across the complete risk period (spanning 15–365 days after antibiotic exposure) may reflect dilution of a stronger temporal association, with a higher UTI risk shortly after antibiotic exposure that attenuates over time. This could potentially explain the highest risk within 30 days after antibiotic exposure, when microbiome recovery remains incomplete.^13^, ^14^, ^15^, ^16^, ^17^^,^^26^ Additionally, higher UTI risk among antibiotic groups such as β-lactams may reflect their greater potential for microbiome disruption.^13^, ^14^, ^15^, ^16^, ^17^

Microbial diversity is central to colonisation resistance; a higher microbial diversity is associated with stronger colonisation resistance against pathogen overgrowth.^26^ Notably, higher UTI risk was observed in men, who are proposed to have lower microbial diversity compared to women, and therefore potentially reduced colonisation resistance.^27^ Consequently, the hypothesized mechanism of microbiome disruption following antibiotic exposure may contribute to explaining the higher UTI risk observed in men. Disruption of the bladder-prostate microbiota may further contribute to UTI risk through local inflammation and epithelial vulnerability.^28^ Among younger men, these mechanisms may be influenced by lifestyle and dietary factors associated with reduced microbial diversity, such as low fibre intake.^27^

Despite lower UTI risk, women had a higher absolute UTI incidence compared to men. Women’s potentially higher microbial diversity may be associated with a smaller proportional loss of colonisation resistance following antibiotic exposure.^27^ Additionally, women may have a greater resistome burden, which may render resident bacteria less susceptible to antibiotic exposure.^29^ Slightly higher UTI risk among premenopausal women, compared to postmenopausal women, may reflect the influence of known UTI risk factors, such as sexual activity and contraceptive use.^3^^,^^4^

These observed sex- and age-based differences in UTI risk following antibiotic exposure may also reflect differences in baseline UTI incidence. Among younger individuals, a lower baseline incidence combined with a larger increase in incidence following antibiotic treatment may result in proportionally higher UTI risk. In contrast, among older individuals, particularly postmenopausal women, a higher baseline incidence combined with smaller increases in incidence following antibiotic treatment may result in a more modest UTI risk. The additional UTI risk attributable to antibiotic exposure may be small relative to other UTI risk factors.

The implications of these findings may extend beyond UTIs, as the hypothesized mechanism of microbiome disruption might not be infection specific. Consequently, these findings could be considered as an additional factor in antibiotic prescribing decisions in primary care, particularly in otherwise healthy individuals. In primary care, RTIs and UTIs account for a large proportion of antibiotic treatments.^7^^,^^8^ For uncomplicated RTIs, antibiotic treatment may provide limited benefit, but may increase adverse events.^8^ While for uncomplicated UTIs, symptomatic treatment (e.g., with analgesics) may offer a safe alternative to immediate antibiotic treatment.^30^ A watchful waiting approach, prescribing antibiotics only if symptoms persist or worsen,^30^ may reduce antibiotic exposure, potentially limiting microbiome disruption^13^, ^14^, ^15^, ^16^, ^17^ and subsequent infection risk.^9^, ^10^, ^11^, ^12^ A translation of the relevant section of the Dutch national guideline on the management of UTIs is provided in Supplementary Material Text 1.^30^

The present study has several strengths. The SCCS design allows individuals to serve as their own control, inherently accounting for time-invariant confounders such as certain UTI risk factors (e.g., urinary tract abnormalities).^20^ This enabled the inclusion of all eligible UTIs and may have mitigated the influence of time-invariant confounders that may be incompletely recorded or absent in routine-care data.^20^^,^^26^ The use of longitudinal data enabled complete follow-up and reliable temporal linkage between antibiotic treatment and UTI, supported by the Dutch healthcare system in which each individual is registered with a single GP practice.^22^ The ELAN database provides broad representation across socio-economic and minority populations, enhancing generalisability.^22^ The large sample size enabled precise estimation of UTI risk across sex, age, and antibiotic groups, including men, who are typically underrepresented in UTI research.^9^, ^10^, ^11^, ^12^

However, there are some limitations. The SCCS design is best suited to transient exposures; consequently, long-term or cumulative effects of repeated antibiotic exposure on UTI risk may be underestimated, with observed associations primarily reflecting short-term influences of antibiotic exposure on UTI risk.^19^ The SCCS design likely mitigated influence from time-invariant confounders, but residual confounding from unmeasured and time-varying confounders such as sexual-, health-care seeking-, and hygiene-related behaviour remains possible.^20^ However, such unmeasured confounding may have been mitigated by the relatively short observation period, during which time-varying confounders are less likely to change substantially. Age, therefore, was treated as time-invariant.

Variability in ICPC-1 coding between GPs may have introduced misclassification. Microbiological confirmation of UTIs was not available in the database. To reduce reliance on individual coding practices, UTIs were identified using a broader, combined approach incorporating both diagnostic and symptom-based ICPC-1 codes. While this approach improved case capture, it may have introduced misclassification due to overlap between symptom-based codes and those of infections such as sexually transmitted infections. However, more than 98% of antibiotic treatments linked to symptom-based ICPC-1 codes corresponded to antibiotics recommended for the treatment of UTIs in Dutch primary care guidelines (e.g., nitrofurantoin),^30^ suggesting any resulting misclassification is likely limited. Additionally, menopausal status was not available in the database and was approximated using an age cutoff based on literature. Although this proxy may have introduced misclassification, the cutoff corresponded to the age at which reporting of menopause-related symptoms increases in Dutch primary care data.^2^

Partial overlap of observation periods resulted in duplicated person-time and UTIs, particularly in antibiotic group stratified analyses, likely reflecting prescription adjustments. Sensitivity analyses excluding such overlaps yielded similar or higher UTI risk estimates compared with the primary analyses, suggesting any resulting bias would have diluted rather than exaggerated the observed associations. As with all observational studies, causality cannot be inferred.^19^^,^^20^ The findings of the present study reflect associations rather than causal effects and should be interpreted with caution. However, the consistent temporal pattern and biological plausibility provide support for a potential underlying association.

To summarise, this large, population-based study observed a modest association between oral antibiotic treatment and UTI incidence in adults, with the highest UTI risk within 30 days and among men ≤65 years old, while women had the highest absolute UTI incidence. These findings may reflect sex- and age-based differences in response to antibiotic treatment^28^^,^^29^ and baseline UTI incidence. However, replication of this study in other settings is warranted to assess whether these associations persist across contexts with differing antibiotic prescribing practices and UTI prevalence, potentially incorporating microbiome profiling to better understand subgroup-specific responses to and recovery dynamics following antibiotic exposure. Future studies might also assess the influence of infection severity, antibiotic dose, treatment duration, or repeated antibiotic exposures on UTI risk. Although the overall increase in UTI risk is small, this increase may still have an impact at the population-level given the high frequency of antibiotic prescribing in primary care. In this context, future studies may help further place the observed findings as an additional factor in antibiotic prescribing decisions in primary care, particularly in otherwise healthy individuals.

## Contributors

MD, WJvD and MS had full access to all the data in the study and take responsibility for the integrity of the data and the accuracy of the data analysis. MD performed the data analysis and drafted the manuscript. WJvD supervised the study design and statistical analysis. MB, HB, and ML contributed to data interpretation and revised the manuscript. MS and CvN conceived the research question and provided guidance and supervision throughout all phases. All authors reviewed and approved the manuscript.

## Data sharing statement

A dataset of coded routine-care data, pseudonymized and extracted from primary care practices into the Extramural LUMC Academic Network (ELAN) data warehouse, was used for this study. These data cannot be shared in an open public repository. Patients provided consent for their data to be reused for dedicated, contextually restricted research and quality management, but not for use in open, publicly accessible domains. Patients can withdraw their data via an informed opt-out procedure; opt-out preferences are digitally recorded in the patient file, and these records are excluded from data sharing. Data are available upon reasonable request through the ELAN data warehouse (elanresearch.nl). The R code used for data management and analysis is available from the corresponding author upon reasonable request.

## Declaration of interests

All authors have completed the ICMJE uniform disclosure form at http://www.icmje.org/disclosure-of-interest/ and declare: no support from any organisation for the submitted work; no financial relationships with any organisations that might have an interest in the submitted work in the previous three years; no other relationships or activities that could appear to have influenced the submitted work.

## Appendix A Supplementary data

Supplementary Material Fig. 1, Text 1, and Tables 1–21

Research Protocol
