# Supplementary material for: Oral antibiotic exposure and urinary tract infection risk in adults: a self-controlled case series study
Source: eClinicalMedicine. 2026 Jul 14;97:104060. doi: 10.1016/j.eclinm.2026.104060 (PMC13382391; doi:10.1016/j.eclinm.2026.104060)
Supplement: Supplementary Material Fig. 1, Text 1, and Tables 1–21 [file mmc1.docx]

**Oral antibiotic exposure and urinary tract infection risk in adults: a self-controlled case series study**

**Supplementary Material**

**Index**

**Figure 1.** Schematic representation of the self-controlled case series study design modelling

**Table 1.** International Classification of Primary Care 1 (ICPC-1) codes excluded during cohort assembly

**Table 2.** Person-time, urinary tract infection events, and incidence rate ratios for the primary analysis

**Table 3.** Sex-and age-stratified incidence rate ratios for the primary analysis

**Table 4.** Antibiotic group stratified incidence rate ratios for the primary analysis

**Table 5.** Sex-, age-, and β-lactam stratified incidence rate ratios for the primary analysis

**Table 6.** Person-time, urinary tract infection events, and incidence rate ratios for the time-adjusted 1 analysis

**Table 7.** Sex-and age-stratified incidence rate ratios for the time-adjusted 1 analysis

**Table 8.** Antibiotic group stratified incidence rate ratios for the time-adjusted 1 analysis

**Table 9.** Sex-, age-, and β-lactam stratified incidence rate ratios for the time-adjusted 1 analysis

**Table 10.** Person-time, urinary tract infection events, and incidence rate ratios for the time-adjusted 2 analysis

**Table 11.** Sex-and age-stratified incidence rate ratios for the time-adjusted 2 analysis

**Table 12.** Antibiotic group stratified incidence rate ratios for the time-adjusted 2 analysis

**Table 13.** Sex-, age-, and β-lactam stratified incidence rate ratios for the time-adjusted 2 analysis

**Table 14.** Person-time, urinary tract infection events, and incidence rate ratios for the censoring analysis

**Table 15.** Sex-and age-stratified incidence rate ratios for the censoring analysis

**Table 16.** Antibiotic group stratified incidence rate ratios for the censoring analysis

**Table 17.** Sex-, age-, and β-lactam stratified incidence rate ratios for the censoring analysis

**Table 18.** Person-time, urinary tract infection events, and incidence rate ratios for the random antibiotic analysis

**Table 19.** Sex-and age-stratified incidence rate ratios for the random antibiotic analysis

**Table 20.** Antibiotic group stratified incidence rate ratios for the random antibiotic analysis

**Table 21.** Sex-, age-, and β-lactam stratified incidence rate ratios for the random antibiotic analysis

**Text 1.** English translation of the Dutch national guideline text on watchful waiting for urinary tract infections

**Figure 1.** **Schematic representation of the self-controlled case series study design modelling**


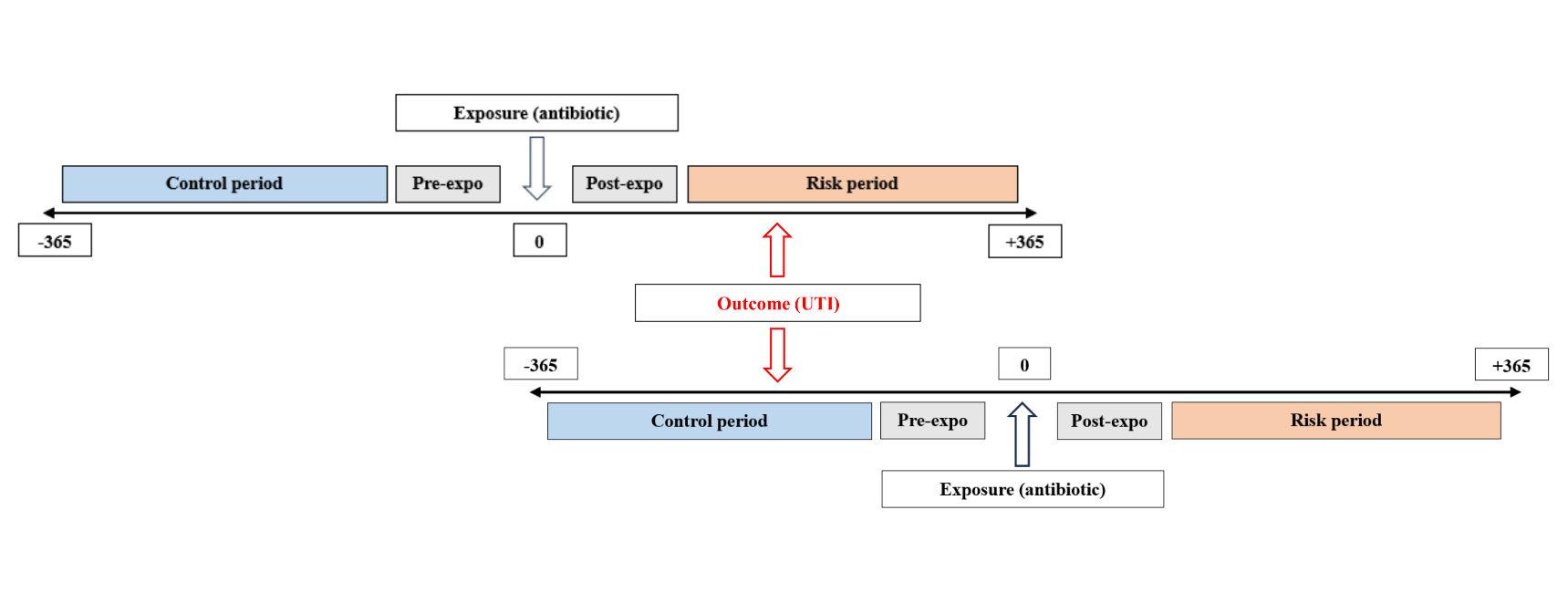


Illustrating two antibiotic treatment (exposure) and one urinary tract infection (UTI; outcome). Control period: 365 to 15 days before antibiotic exposure (blue). Risk period: 15 to 365 days after antibiotic exposure (orange). Pre-exposure period: 14 to 1 days before antibiotic exposure (grey). Post-exposure period: 1 to 14 days after antibiotic exposure (grey). Due to the modelling structure, a single UTI can occur in the 365 after a first (i.e. risk period), as well as in the 365 days before a second (i.e. control period) antibiotic exposure.

**Table 1.** **International Classification of Primary Care 1 (ICPC-1) codes excluded during cohort assembly**

| **ICPC-1 code** | **Description** | **n** |
| --- | --- | --- |
| L82 | Congenital anomalies of the musculoskeletal system | 1,504 |
| N81 | Other nervous system injuries | 1,098 |
| N85 | Congenital anomalies of the nervous system | 533 |
| N99 | Other diseases of the nervous system | 3,355 |
| T90 | Diabetes Mellitus | 74,109 |
| T99 | Other diseases of the endocrine glands, metabolism, or nutrition | 7,687 |
| W78 | Pregnancy confirmed | 26,777 |
| W90 | Normal delivery live birth | 20,675 |
| W91 | Normal delivery stillbirth | 239 |
| W92 | Complicated delivery live birth | 4,600 |
| W93 | Complicated delivery stillbirth | 310 |
| X14 | Vaginal discharge | 13,749 |
| X15 | Other complaints of the vagina | 13,950 |
| X72 | Vaginal candidiasis | 21,571 |
| X75 | Malignancy of the cervix uteri | 1,164 |
| X77 | Other malignancy of the female genital organs | 2,463 |
| X78 | Benign neoplasm of the uterus or cervix uteri | 4,851 |
| X84.02 | Bacterial vaginosis | 7,355 |
| X87 | Prolapse of the vagina or uterus | 10,702 |
| Y06 | Prostate complaints | 5,655 |
| Y85 | Benign prostatic hypertrophy | 11,201 |
| U04 | Urine incontinence | 21,937 |
| U05.02 | Urine retention | 6,895 |
| U28 | Functional disorder of the urinary tract | 1,304 |
| U75 | Malignancy of the kidney | 1,575 |
| U76 | Malignancy of the bladder | 3,669 |
| U78 | Benign neoplasm of the urinary tract | 308 |
| U79 | Unspecified neoplasm of the urinary tract | 522 |
| U80 | Injury to the urinary tract | 116 |
| U85 | Congenital anomalies of the urinary tract | 545 |
| U88 | Glomerulonephritis or nephrosis | 778 |
| U95 | Urolithiasis | 12,332 |
| U98.02 | Glucosuria | 1,178 |
| U99 | Other diseases of the urinary tract | 34,053 |

**Table 2.** **Person-time, urinary tract infection events, and incidence rate ratios for the primary analysis**

| **Time period*** | **Person-time (years)**** | **UTI events (n)** | **IR (per PY)***** | **IRR (95% CI)** |
| --- | --- | --- | --- | --- |
| Control period (-365 to -15 days) | 422,656.85 | 458,742 | 1.08 | Reference |
| Risk period 1 (+15 to +30 days) | 19,266.41 | 36,474 | 1.89 | 1.74 (1.73 – 1.76) |
| Risk period 2 (+15 to +90 days) | 91,515.44 | 129,508 | 1.42 | 1.30 (1.30 – 1.31) |
| Risk period 3 (+15 to +180 days) | 199,889.00 | 248,922 | 1.25 | 1.15 (1.14 – 1.15) |
| Risk period 4 (+15 to +365 days) | 422,656.85 | 472,446 | 1.12 | 1.03 (1.03 – 1.04) |

The primary analyses include all antibiotic exposures per individual.

*Time period: time period before (i.e. control period) or after (i.e. risk period 1, risk period 2, risk period 3, risk period 4) antibiotic exposure (in days).

**Person-time: total time contributed to each time period (in years).

***Per PY: per person-year.

IRR indicates incidence rate ratio; CI, confidence interval.

**Table 3.** **Sex-and age-stratified incidence rate ratios for the primary analysis**

| **Subgroup** | **Time period*** | **Person-time (years)**** | **UTI events (n)** | **IR (per PY)***** | **IRR (95% CI)** |
| --- | --- | --- | --- | --- | --- |
| Women ≤50 years | Control period  (-365 to -15 days) | 105,784.34 | 76,524 | 0.72 | Reference |
|  | Risk period 1  (+15 to +30 days) | 4,822.08 | 6,888 | 1.43 | 1.98 (1.93 – 2.02) |
|  | Risk period 2  (+15 to +90 days) | 22,904.87 | 23,694 | 1.03 | 1.43 (1.41 – 1.45) |
|  | Risk period 3  (+15 to +180 days) | 50,029.06 | 45,784 | 0.92 | 1.27 (1.25 – 1.28) |
|  | Risk period 4  (+15 to +365 days) | 105,784.34 | 87,363 | 0.83 | 1.14 (1.13 – 1.15) |
|  |  |  |  |  |  |
| Women >50 years | Control period  (-365 to -15 days) | 244,597.75 | 322,530 | 1.32 | Reference |
|  | Risk period 1  (+15 to +30 days) | 11,149.75 | 22,356 | 2.01 | 1.52 (1.50 – 1.54) |
|  | Risk period 2  (+15 to +90 days) | 52,961.34 | 83,857 | 1.58 | 1.20 (1.19 – 1.21) |
|  | Risk period 3  (+15 to +180 days) | 115,678.71 | 164,814 | 1.43 | 1.08 (1.07 – 1.09) |
|  | Risk period 4  (+15 to +365 days) | 244,597.75 | 319,246 | 1.31 | 0.99 (0.99 – 1.00) |
|  |  |  |  |  |  |
| Men ≤65 years | Control period  (-365 to -15 days) | 21,122.46 | 13,295 | 0.63 | Reference |
|  | Risk period 1  (+15 to +30 days) | 962.85 | 2,103 | 2.18 | 3.47 (3.31 – 3.63) |
|  | Risk period 2  (+15 to +90 days) | 4,573.53 | 5,697 | 1.25 | 1.98 (1.92 – 2.04) |
|  | Risk period 3  (+15 to +180 days) | 9,989.54 | 9,651 | 0.97 | 1.54 (1.50 – 1.58) |
|  | Risk period 4  (+15 to +365 days) | 21,122.46 | 16,568 | 0.78 | 1.25 (1.22 – 1.28) |
|  |  |  |  |  |  |
| Men >65 years | Control period  (-365 to -15 days) | 51,152.30 | 46,393 | 0.91 | Reference |
|  | Risk period 1  (+15 to +30 days) | 2,331.73 | 5,127 | 2.20 | 2.42 (2.36 – 2.50) |
|  | Risk period 2  (+15 to +90 days) | 11,075.71 | 16,260 | 1.47 | 1.62 (1.59 – 1.65) |
|  | Risk period 3  (+15 to +180 days) | 24,191.69 | 28,673 | 1.19 | 1.30 (1.29 – 1.33) |
|  | Risk period 4  (+15 to +365 days) | 51,152.30 | 49,269 | 0.96 | 1.06 (1.05 – 1.08) |

The primary analyses include all antibiotic exposures per individual.

*Time period: time period before (i.e. control period) or after (i.e. risk period 1, risk period 2, risk period 3, risk period 4) antibiotic exposure (in days).

**Person-time: total time contributed to each time period (in years).

***Per PY: per person-year.

IRR indicates incidence rate ratio; CI, confidence interval.

**Table 4.** **Antibiotic group stratified incidence rate ratios for the primary analysis**

| **Subgroup** | **Time period*** | **Person-time (years)**** | **UTI events (n)** | **IR (per PY)***** | **IRR (95% CI)** |
| --- | --- | --- | --- | --- | --- |
| β-lactams | Control period  (-365 to -15 days) | 79,026.65 | 72,601 | 0.92 | Reference |
|  | Risk period 1  (+15 to +30 days) | 2,602.36 | 6,476 | 1.80 | 1.96 (1.91 – 2.01) |
|  | Risk period 2  (+15 to +90 days) | 17,111.18 | 22,132 | 1.29 | 1.41 (1.39 – 1.43) |
|  | Risk period 3  (+15 to +180 days) | 37,374.43 | 41,758 | 1.12 | 1.22 (1.20 – 1.23) |
|  | Risk period 4  (+15 to +365 days) | 79,026.65 | 79,101 | 1.00 | 1.09 (1.08 – 1.10) |
|  |  |  |  |  |  |
| Macrolides | Control period  (-365 to -15 days) | 23,758.45 | 21,750 | 0.92 | Reference |
|  | Risk period 1  (+15 to +30 days) | 1,083.01 | 1,111 | 1.03 | 1.12 (1.06 – 1.19) |
|  | Risk period 2  (+15 to +90 days) | 5,144.28 | 5,249 | 1.02 | 1.12 (1.08 – 1.15) |
|  | Risk period 3  (+15 to +180 days) | 11,236.19 | 11,423 | 1.02 | 1.11 (1.09 – 1.14) |
|  | Risk period 4  (+15 to +365 days) | 23,758.45 | 24,022 | 1.01 | 1.10 (1.08 – 1.13) |
|  |  |  |  |  |  |
| Other antibacterials**** | Control period  (-365 to -15 days) | 230,641.36 | 271,352 | 1.18 | Reference |
|  | Risk period 1  (+15 to +30 days) | 10,513.57 | 21,583 | 2.05 | 1.75 (1.72 – 1.77) |
|  | Risk period 2  (+15 to +90 days) | 49,939.44 | 76,251 | 1.53 | 1.30 (1.29 – 1.31) |
|  | Risk period 3  (+15 to +180 days) | 109,078.25 | 145,604 | 1.34 | 1.14 (1.13 – 1.14) |
|  | Risk period 4  (+15 to +365 days) | 230,641.36 | 274,630 | 1.19 | 1.01 (1.01 – 1.02) |
|  |  |  |  |  |  |
| Quinolones | Control period  (-365 to -15 days) | 48,670.08 | 46,083 | 0.95 | Reference |
|  | Risk period 1  (+15 to +30 days) | 2,218.58 | 4,626 | 2.09 | 2.20 (2.14 – 2.27) |
|  | Risk period 2  (+15 to +90 days) | 10,538.25 | 14,840 | 1.41 | 1.49 (1.46 – 1.52) |
|  | Risk period 3  (+15 to +180 days) | 23,017.76 | 27,706 | 1.20 | 1.27 (1.25 – 1.29) |
|  | Risk period 4  (+15 to +365 days) | 48,670.08 | 50,494 | 1.04 | 1.10 (1.08 – 1.11) |
|  |  |  |  |  |  |
| Sulphonamides | Control period  (-365 to -15 days) | 28,788.25 | 47,214 | 1.64 | Reference |
|  | Risk period 1  (+15 to +30 days) | 1,312.29 | 2,988 | 2.28 | 1.39 (1.34 – 1.44) |
|  | Risk period 2  (+15 to +90 days) | 6,233.35 | 11,456 | 1.84 | 1.12 (1.10 – 1.14) |
|  | Risk period 3  (+15 to +180 days) | 13,614.95 | 22,420 | 1.65 | 1.00 (0.99 – 1.02) |
|  | Risk period 4  (+15 to +365 days) | 28,788.25 | 42,155 | 1.46 | 0.89 (0.88 – 0.91) |
|  |  |  |  |  |  |
| Tetracyclines | Control period  (-365 to -15 days) | 18,064.61 | 15,370 | 0.85 | Reference |
|  | Risk period 1  (+15 to +30 days) | 823.46 | 803 | 0.98 | 1.15 (1.07 – 1.23) |
|  | Risk period 2  (+15 to +90 days) | 3,911.43 | 3,702 | 0.95 | 1.11 (1.07 – 1.15) |
|  | Risk period 3  (+15 to +180 days) | 8,543.38 | 8,188 | 0.96 | 1.13 (1.10 – 1.16) |
|  | Risk period 4  (+15 to +365 days) | 18,064.61 | 17,368 | 0.96 | 1.13 (1.11 – 1.16) |

The primary analyses include all antibiotic exposures per individual.

*Time period: time period before (i.e. control period) or after (i.e. risk period 1, risk period 2, risk period 3, risk period 4) antibiotic exposure (in days).

**Person-time: total time contributed to each time period (in years).

***Per PY: per person-year.

****Other antibacterials: antibiotics included in the ATC subgroup J01XX ‘’other antibacterials’’.

IRR indicates incidence rate ratio; CI, confidence interval.

| **Subgroup** | **Time period*** | **Person-time (years)**** | **UTI events (n)** | **IR (per PY)***** | **IRR (95% CI)** |
| --- | --- | --- | --- | --- | --- |
| Women ≤50 years | Control period  (-365 to -15 days) | 20,605.45 | 16,214 | 0.79 | Reference |
|  | Risk period 1  (+15 to +30 days) | 939.28 | 1,202 | 1.28 | 1.63 (1.53 – 1.72) |
|  | Risk period 2  (+15 to +90 days) | 4,461.58 | 4,451 | 1.00 | 1.27 (1.23 – 1.31) |
|  | Risk period 3  (+15 to +180 days) | 9,745.03 | 8,831 | 0.91 | 1.15 (1.12 – 1.18) |
|  | Risk period 4  (+15 to +365 days) | 20,605.45 | 17,394 | 0.84 | 1.07 (1.05 – 1.10) |
|  |  |  |  |  |  |
| Women >50 years | Control period  (-365 to -15 days) | 38,693.13 | 41,281 | 1.07 | Reference |
|  | Risk period 1  (+15 to +30 days) | 1,763.79 | 3,398 | 1.93 | 1.81 (1.74 – 1.87) |
|  | Risk period 2  (+15 to +90 days) | 8,378.00 | 11,758 | 1.40 | 1.32 (1.29 – 1.34) |
|  | Risk period 3  (+15 to +180 days) | 18,299.31 | 22,526 | 1.23 | 1.15 (1.14 – 1.17) |
|  | Risk period 4  (+15 to +365 days) | 38,693.13 | 43,470 | 1.12 | 1.05 (1.04 – 1.07) |
|  |  |  |  |  |  |
| Men ≤65 years | Control period  (-365 to -15 days) | 5,710.18 | 3,706 | 0.65 | Reference |
|  | Risk period 1  (+15 to +30 days) | 260.29 | 530 | 2.04 | 3.14 (2.86 – 3.43) |
|  | Risk period 2  (+15 to +90 days) | 1,236.39 | 1,605 | 1.30 | 2.00 (1.89 – 2.12) |
|  | Risk period 3  (+15 to +180 days) | 2,700.54 | 2,751 | 1.02 | 1.57 (1.49 – 1.65) |
|  | Risk period 4  (+15 to +365 days) | 5,710.18 | 4,935 | 0.86 | 1.33 (1.28 – 1.39) |
|  |  |  |  |  |  |
| Men >65 years | Control period  (-365 to -15 days) | 14,017.90 | 11,400 | 0.81 | Reference |
|  | Risk period 1  (+15 to +30 days) | 638.99 | 1,346 | 2.11 | 2.59 (2.45 – 2.74) |
|  | Risk period 2  (+15 to +90 days) | 3,035.21 | 4,318 | 1.42 | 1.75 (1.69 – 1.81) |
|  | Risk period 3  (+15 to +180 days) | 6,629.55 | 7,650 | 1.15 | 1.42 (1.38 – 1.46) |
|  | Risk period 4  (+15 to +365 days) | 14,017.90 | 13,302 | 0.95 | 1.17 (1.14 – 1.20) |

**Table 5. Sex-, age-, and β-lactam stratified incidence rate ratios for the primary analysis**

The primary analyses include all antibiotic exposures per individual.

*Time period: time period before (i.e. control period) or after (i.e. risk period 1, risk period 2, risk period 3, risk period 4) antibiotic exposure (in days).

**Person-time: total time contributed to each time period (in years).

***Per PY: per person-year.

IRR indicates incidence rate ratio; CI, confidence interval.

| **Time period*** | **Person-time (years)**** | **UTI events (n)** | **IR (per PY)***** | **IRR (95% CI)** |
| --- | --- | --- | --- | --- |
| Control period (-365 to -31 days) | 403,390.44 | 429,551 | 1.07 | Reference |
| Risk period 1 (+31 to +90 days) | 72,249.03 | 93,034 | 1.29 | 1.21 (1.20 – 1.22) |
| Risk period 2 (+31 to +180 days) | 180,622.59 | 212,448 | 1.18 | 1.11 (1.10 – 1.11) |
| Risk period 3 (+31 to +365 days) | 403,390.44 | 435,972 | 1.08 | 1.02 (1.01 – 1.02) |

**Table 6. Person-time, urinary tract infection events, and incidence rate ratios for the time-adjusted 1 analysis**

The time-adjusted 1 analyses include an extended 30-day pre-and post-exposure period.

*Time period: time period before (i.e. control period) or after (i.e. risk period 1, risk period 2, risk period 3) antibiotic exposure (in days).

**Person-time: total time contributed to each time period (in years).

***Per PY: per person-year.

IRR indicates incidence rate ratio; CI, confidence interval.

**Table 7.** **Sex-and age-stratified incidence rate ratios for the time-adjusted 1 analysis**

| **Subgroup** | **Time period*** | **Person-time (years)**** | **UTI events (n)** | **IR (per PY)***** | **IRR (95% CI)** |
| --- | --- | --- | --- | --- | --- |
| Women ≤50 years | Control period  (-365 to -31 days) | 100,962.26 | 71,303 | 0.71 | Reference |
|  | Risk period 1  (+31 to +90 days) | 18,082.79 | 16,806 | 0.93 | 1.32 (1.29 – 1.34) |
|  | Risk period 2  (+31 to +180 days) | 45,206.98 | 38,896 | 0.86 | 1.22 (1.20 – 1.23) |
|  | Risk period 3  (+31 to +365 days) | 100,962.26 | 80,475 | 0.80 | 1.13 (1.12 – 1.14) |
|  |  |  |  |  |  |
| Women >50 years | Control period  (-365 to -31 days) | 233,447.99 | 303,579 | 1.30 | Reference |
|  | Risk period 1  (+31 to +90 days) | 41,811.58 | 61,501 | 1.47 | 1.13 (1.12 – 1.14) |
|  | Risk period 2  (+31 to +180 days) | 104,528.95 | 142,458 | 1.36 | 1.05 (1.04 – 1.06) |
|  | Risk period 3  (+31 to +365 days) | 233,447.99 | 296,890 | 1.27 | 0.98 (0.97 – 0.98) |
|  |  |  |  |  |  |
| Men ≤65 years | Control period  (-365 to -31 days) | 20,159.62 | 11,974 | 0.59 | Reference |
|  | Risk period 1  (+31 to +90 days) | 3,610.68 | 3,594 | 1.00 | 1.68 (1.62 – 1.74) |
|  | Risk period 2  (+31 to +180 days) | 9,026.69 | 7,548 | 0.84 | 1.41 (1.37 – 1.45) |
|  | Risk period 3  (+31 to +365 days) | 20,159.62 | 14,465 | 0.72 | 1.21 (1.18 – 1.24) |
|  |  |  |  |  |  |
| Men >65 years | Control period  (-365 to -31 days) | 48,820.58 | 42,695 | 0.88 | Reference |
|  | Risk period 1  (+31 to +90 days) | 8,743.98 | 11,133 | 1.27 | 1.46 (1.43 – 1.49) |
|  | Risk period 2  (+31 to +180 days) | 21,859.96 | 23,546 | 1.08 | 1.23 (1.21 – 1.25) |
|  | Risk period 3  (+31 to +365 days) | 48,820.58 | 44,142 | 0.90 | 1.03 (1.02 – 1.05) |

The time-adjusted 1 analyses include an extended 30-day pre-and post-exposure period.

*Time period: time period before (i.e. control period) or after (i.e. risk period 1, risk period 2, risk period 3) antibiotic exposure (in days).

**Person-time: total time contributed to each time period (in years).

***Per PY: per person-year.

IRR indicates incidence rate ratio; CI, confidence interval.

**Table 8.** **Antibiotic group stratified incidence rate ratios for the time-adjusted 1 analysis**

| **Subgroup** | **Time period*** | **Person-time (years)**** | **UTI events (n)** | **IR (per PY)***** | **IRR (95% CI)** |
| --- | --- | --- | --- | --- | --- |
| β-lactams | Control period  (-365 to -31 days) | 75,424.30 | 68,057 | 0.90 | Reference |
|  | Risk period 1  (+31 to +90 days) | 13,508.83 | 15,656 | 1.16 | 1.28 (1.26 – 1.31) |
|  | Risk period 2  (+31 to +180 days) | 33,772.07 | 35,282 | 1.05 | 1.16 (1.14 – 1.17) |
|  | Risk period 3  (+31 to +365 days) | 75,424.30 | 72,625 | 0.96 | 1.07 (1.06 – 1.08) |
|  |  |  |  |  |  |
| Macrolides | Control period  (-365 to -31 days) | 22,675.44 | 20,620 | 0.91 | Reference |
|  | Risk period 1  (+31 to +90 days) | 4,061.27 | 4,138 | 1.02 | 1.12 (1.08 – 1.16) |
|  | Risk period 2  (+31 to +180 days) | 10,153.18 | 10,312 | 1.02 | 1.12 (1.09 – 1.14) |
|  | Risk period 3  (+31 to +365 days) | 22,675.44 | 22,911 | 1.01 | 1.11 (1.09 – 1.13) |
|  |  |  |  |  |  |
| Other antibacterials**** | Control period  (-365 to -31 days) | 220,127.79 | 255,411 | 1.16 | Reference |
|  | Risk period 1  (+31 to +90 days) | 39,425.87 | 54,668 | 1.39 | 1.20 (1.18 – 1.21) |
|  | Risk period 2  (+31 to +180 days) | 98,564.68 | 124,021 | 1.26 | 1.08 (1.08 – 1.09) |
|  | Risk period 3  (+31 to +365 days) | 220,127.79 | 253,047 | 1.15 | 0.99 (0.99 – 1.00) |
|  |  |  |  |  |  |
| Quinolones | Control period  (-365 to -31 days) | 46,451.50 | 41,720 | 0.90 | Reference |
|  | Risk period 1  (+31 to +90 days) | 8,319.67 | 10,214 | 1.23 | 1.37 (1.34 – 1.40) |
|  | Risk period 2  (+31 to +180 days) | 20,799.18 | 23,080 | 1.11 | 1.24 (1.22 – 1.26) |
|  | Risk period 3  (+31 to +365 days) | 46,451.50 | 45,868 | 0.99 | 1.10 (1.09 – 1.11) |
|  |  |  |  |  |  |
| Sulphonamides | Control period  (-365 to -31 days) | 27,475.96 | 43,707 | 1.59 | Reference |
|  | Risk period 1  (+31 to +90 days) | 4,921.07 | 8,468 | 1.72 | 1.08 (1.06 – 1.11) |
|  | Risk period 2  (+31 to +180 days) | 12,302.67 | 19,432 | 1.58 | 0.99 (0.98 – 1.01) |
|  | Risk period 3  (+31 to +365 days) | 27,475.96 | 39,167 | 1.43 | 0.90 (0.88 – 0.91) |
|  |  |  |  |  |  |
| Tetracyclines | Control period  (-365 to -31 days) | 17,241.15 | 14,628 | 0.85 | Reference |
|  | Risk period 1  (+31 to +90 days) | 3,087.97 | 2,899 | 0.94 | 1.11 (1.06 – 1.15) |
|  | Risk period 2  (+31 to +180 days) | 7,719.92 | 7,285 | 0.96 | 1.13 (1.10 – 1.16) |
|  | Risk period 3  (+31 to +365 days) | 17,241.15 | 16,565 | 0.96 | 1.13 (1.11 – 1.16) |

The time-adjusted 1 analyses include an extended 30-day pre-and post-exposure period.

*Time period: time period before (i.e. control period) or after (i.e. risk period 1, risk period 2, risk period 3) antibiotic exposure (in days).

**Person-time: total time contributed to each time period (in years).

***Per PY: per person-year.

****Other antibacterials: antibiotics included in the ATC subgroup J01XX ‘’other antibacterials’’.

IRR indicates incidence rate ratio; CI, confidence interval.

**Table 9. Sex-, age-, and β-lactam stratified incidence rate ratios for the time-adjusted 1 analysis**

| **Subgroup** | **Time period*** | **Person-time (years)**** | **UTI events (n)** | **IR (per PY)***** | **IRR (95% CI)** |
| --- | --- | --- | --- | --- | --- |
| Women ≤50 years | Control period  (-365 to -31 days) | 19,666.17 | 15,260 | 0.78 | Reference |
|  | Risk period 1  (+31 to +90 days) | 3,522.30 | 3,249 | 0.92 | 1.19 (1.15 – 1.24) |
|  | Risk period 2  (+31 to +180 days) | 8,805.75 | 7,629 | 0.87 | 1.12 (1.09 – 1.15) |
|  | Risk period 3  (+31 to +365 days) | 19,666.17 | 16,192 | 0.82 | 1.06 (1.04 – 1.09) |
|  |  |  |  |  |  |
| Women >50 years | Control period  (-365 to -31 days) | 36,929.34 | 38,905 | 1.05 | Reference |
|  | Risk period 1  (+31 to +90 days) | 6,614.21 | 8,360 | 1.26 | 1.20 (1.17 – 1.23) |
|  | Risk period 2  (+31 to +180 days) | 16,535.52 | 19,128 | 1.16 | 1.10 (1.08 – 1.11) |
|  | Risk period 3  (+31 to +365 days) | 36,929.34 | 40,072 | 1.09 | 1.03 (1.02 – 1.05) |
|  |  |  |  |  |  |
| Men ≤65 years | Control period  (-365 to -31 days) | 5,449.88 | 3,424 | 0.63 | Reference |
|  | Risk period 1  (+31 to +90 days) | 976.10 | 1,075 | 1.10 | 1.75 (1.64 – 1.88) |
|  | Risk period 2  (+31 to +180 days) | 2,440.25 | 2,221 | 0.91 | 1.45 (1.37 – 1.53) |
|  | Risk period 3  (+31 to +365 days) | 5,449.88 | 4,405 | 0.81 | 1.29 (1.23 – 1.35) |
|  |  |  |  |  |  |
| Men >65 years | Control period  (-365 to -31 days) | 13,378.91 | 10,468 | 0.78 | Reference |
|  | Risk period 1  (+31 to +90 days) | 2,396.22 | 2,972 | 1.24 | 1.59 (1.52 – 1.65) |
|  | Risk period 2  (+31 to +180 days) | 5,990.55 | 6,304 | 1.05 | 1.35 (1.30 – 1.39) |
|  | Risk period 3  (+31 to +365 days) | 13,378.91 | 11,956 | 0.89 | 1.14 (1.11 – 1.17) |

The time-adjusted 1 analyses include an extended 30-day pre-and post-exposure period.

*Time period: time period before (i.e. control period) or after (i.e. risk period 1, risk period 2, risk period 3) antibiotic exposure (in days).

**Person-time: total time contributed to each time period (in years).

***Per PY: per person-year.

IRR indicates incidence rate ratio; CI, confidence interval.

**Table 10. Person-time, urinary tract infection events, and incidence rate ratios for the time-adjusted 2 analysis**

| **Time period*** | **Person-time (years)**** | **UTI events (n)** | **IR (per PY)***** | **IRR (95% CI)** |
| --- | --- | --- | --- | --- |
| Control period (-180 to -15 days) | 159,974.01 | 235,492 | 1.47 | Reference |
| Risk period 1 (+15 to +30 days) | 15,419.18 | 36,474 | 2.37 | 1.61 (1.59 – 1.63) |
| Risk period 2 (+15 to +90 days) | 73,241.11 | 129,508 | 1.77 | 1.20 (1.19 – 1.21) |
| Risk period 3 (+15 to +180 days) | 159,974.01 | 248,922 | 1.56 | 1.06 (1.05 – 1.06) |

The time-adjusted 2 analyses include a shortened 180-day observation period.

*Time period: time period before (i.e. control period) or after (i.e. risk period 1, risk period 2, risk period 3) antibiotic exposure (in days).

**Person-time: total time contributed to each time period (in years).

***Per PY: per person-year.

IRR indicates incidence rate ratio; CI, confidence interval.

**Table 11.** **Sex-and age-stratified incidence rate ratios for the time-adjusted 2 analysis**

| **Subgroup** | **Time period*** | **Person-time (years)**** | **UTI events (n)** | **IR (per PY)***** | **IRR (95% CI)** |
| --- | --- | --- | --- | --- | --- |
| Women ≤50 years | Control period  (-180 to -15 days) | 40,267.21 | 40,384 | 1.00 | Reference |
|  | Risk period 1  (+15 to +30 days) | 3,881.18 | 6,888 | 1.78 | 1.77 (1.73 – 1.82) |
|  | Risk period 2  (+15 to +90 days) | 18,435.59 | 23,694 | 1.29 | 1.28 (1.26 – 1.30) |
|  | Risk period 3  (+15 to +180 days) | 40,267.21 | 45,784 | 1.14 | 1.13 (1.12 – 1.15) |
|  |  |  |  |  |  |
| Women >50 years | Control period  (-180 to -15 days) | 92,772.75 | 161,981 | 1.75 | Reference |
|  | Risk period 1  (+15 to +30 days) | 8,941.95 | 22,356 | 2.50 | 1.43 (1.41 – 1.45) |
|  | Risk period 2  (+15 to +90 days) | 42,474.27 | 83,857 | 1.97 | 1.13 (1.12 – 1.14) |
|  | Risk period 3  (+15 to +180 days) | 92,772.75 | 164,814 | 1.78 | 1.02 (1.01 – 1.03) |
|  |  |  |  |  |  |
| Men ≤65 years | Control period  (-180 to -15 days) | 7,913.92 | 7,400 | 0.94 | Reference |
|  | Risk period 1  (+15 to +30 days) | 762.79 | 2,103 | 2.76 | 2.95 (2.81 – 3.10) |
|  | Risk period 2  (+15 to +90 days) | 3,623.24 | 5,697 | 1.57 | 1.68 (1.62 – 1.74) |
|  | Risk period 3  (+15 to +180 days) | 7,913.92 | 9,651 | 1.22 | 1.30 (1.27 – 1.34) |
|  |  |  |  |  |  |
| Men >65 years | Control period  (-180 to -15 days) | 19,020.12 | 25,727 | 1.35 | Reference |
|  | Risk period 1  (+15 to +30 days) | 1,833.27 | 5,127 | 2.80 | 2.07 (2.01 – 2.13) |
|  | Risk period 2  (+15 to +90 days) | 8,708.01 | 16,260 | 1.87 | 1.38 (1.35 – 1.41) |
|  | Risk period 3  (+15 to +180 days) | 19,020.12 | 28,673 | 1.51 | 1.12 (1.10 – 1.13) |

The time-adjusted 2 analyses include a shortened 180-day observation period.

*Time period: time period before (i.e. control period) or after (i.e. risk period 1, risk period 2, risk period 3) antibiotic exposure (in days).

**Person-time: total time contributed to each time period (in years).

***Per PY: per person-year.

IRR indicates incidence rate ratio; CI, confidence interval.

**Table 12.** **Antibiotic group stratified incidence rate ratios for the time-adjusted 2 analysis**

| **Subgroup** | **Time period*** | **Person-time (years)**** | **UTI events (n)** | **IR (per PY)***** | **IRR (95% CI)** |
| --- | --- | --- | --- | --- | --- |
| β-lactams | Control period  (-180 to -15 days) | 25,412.43 | 36,999 | 1.46 | Reference |
|  | Risk period 1  (+15 to +30 days) | 2,449.39 | 6,476 | 2.64 | 1.82 (1.77 – 1.87) |
|  | Risk period 2  (+15 to +90 days) | 11,634.61 | 22,132 | 1.90 | 1.31 (1.29 – 1.33) |
|  | Risk period 3  (+15 to +180 days) | 25,412.43 | 41,758 | 1.64 | 1.13 (1.11 – 1.15) |
|  |  |  |  |  |  |
| Macrolides | Control period  (-180 to -15 days) | 6,784.07 | 10,724 | 1.58 | Reference |
|  | Risk period 1  (+15 to +30 days) | 653.89 | 1,111 | 1.70 | 1.08 (1.01 – 1.14) |
|  | Risk period 2  (+15 to +90 days) | 3,105.96 | 5,249 | 1.69 | 1.07 (1.03 – 1.11) |
|  | Risk period 3  (+15 to +180 days) | 6,784.07 | 11,423 | 1.68 | 1.07 (1.04 – 1.10) |
|  |  |  |  |  |  |
| Other antibacterials**** | Control period  (-180 to -15 days) | 94,220.28 | 137,825 | 1.46 | Reference |
|  | Risk period 1  (+15 to +30 days) | 9,081.47 | 21,583 | 2.38 | 1.63 (1.60 – 1.65) |
|  | Risk period 2  (+15 to +90 days) | 43,137.00 | 76,251 | 1.77 | 1.21 (1.20 – 1.22) |
|  | Risk period 3  (+15 to +180 days) | 94,220.28 | 145,604 | 1.55 | 1.06 (1.05 – 1.06) |
|  |  |  |  |  |  |
| Quinolones | Control period  (-180 to -15 days) | 19,687.31 | 25,674 | 1.30 | Reference |
|  | Risk period 1  (+15 to +30 days) | 1,897.57 | 4,626 | 2.44 | 1.87 (1.81 – 1.93) |
|  | Risk period 2  (+15 to +90 days) | 9,013.47 | 14,840 | 1.65 | 1.26 (1.24 – 1.29) |
|  | Risk period 3  (+15 to +180 days) | 19,687.31 | 27,706 | 1.41 | 1.08 (1.06 – 1.10) |
|  |  |  |  |  |  |
| Sulphonamides | Control period  (-180 to -15 days) | 11,063.03 | 24,897 | 2.25 | Reference |
|  | Risk period 1  (+15 to +30 days) | 1,066.32 | 2,988 | 2.80 | 1.25 (1.20 – 1.29) |
|  | Risk period 2  (+15 to +90 days) | 5,065.00 | 11,456 | 2.26 | 1.01 (0.98 – 1.03) |
|  | Risk period 3  (+15 to +180 days) | 11,063.03 | 22,420 | 2.03 | 0.90 (0.88 – 0.92) |
|  |  |  |  |  |  |
| Tetracyclines | Control period  (-180 to -15 days) | 5,084.76 | 7,615 | 1.50 | Reference |
|  | Risk period 1  (+15 to +30 days) | 490.10 | 803 | 1.64 | 1.09 (1.02 – 1.18) |
|  | Risk period 2  (+15 to +90 days) | 2,327.96 | 3,702 | 1.59 | 1.06 (1.02 – 1.10) |
|  | Risk period 3  (+15 to +180 days) | 5,084.76 | 8,188 | 1.61 | 1.08 (1.04 – 1.11) |

The time-adjusted 2 analyses include a shortened 180-day observation period.

*Time period: time period before (i.e. control period) or after (i.e. risk period 1, risk period 2, risk period 3) antibiotic exposure (in days).

**Person-time: total time contributed to each time period (in years).

***Per PY: per person-year.

****Other antibacterials: antibiotics included in the ATC subgroup J01XX ‘’other antibacterials’’.

IRR indicates incidence rate ratio; CI, confidence interval.

**Table 13. Sex-, age-, and β-lactam stratified incidence rate ratios for the time-adjusted 2 analysis**

| **Subgroup** | **Time period*** | **Person-time (years)**** | **UTI events (n)** | **IR (per PY)***** | **IRR (95% CI)** |
| --- | --- | --- | --- | --- | --- |
| Women ≤50 years | Control period  (-180 to -15 days) | 6,259.14 | 8,306 | 1.33 | Reference |
|  | Risk period 1  (+15 to +30 days) | 603.29 | 1,202 | 1.99 | 1.50 (1.41 – 1.60) |
|  | Risk period 2  (+15 to +90 days) | 2,865.63 | 4,451 | 1.55 | 1.17 (1.13 – 1.21) |
|  | Risk period 3  (+15 to +180 days) | 6,259.14 | 8,831 | 1.41 | 1.06 (1.03 – 1.10) |
|  |  |  |  |  |  |
| Women >50 years | Control period  (-180 to -15 days) | 12,543.28 | 20,569 | 1.64 | Reference |
|  | Risk period 1  (+15 to +30 days) | 1,208.99 | 3,398 | 2.81 | 1.71 (1.65 – 1.78) |
|  | Risk period 2  (+15 to +90 days) | 5,742.71 | 11,758 | 2.05 | 1.25 (1.22 – 1.28) |
|  | Risk period 3  (+15 to +180 days) | 12,543.28 | 22,526 | 1.80 | 1.10 (1.08 – 1.12) |
|  |  |  |  |  |  |
| Men ≤65 years | Control period  (-180 to -15 days) | 1,815.66 | 1,857 | 1.02 | Reference |
|  | Risk period 1  (+15 to +30 days) | 175.00 | 530 | 3.03 | 2.96 (2.69 – 3.26) |
|  | Risk period 2  (+15 to +90 days) | 831.27 | 1,605 | 1.93 | 1.89 (1.77 – 2.02) |
|  | Risk period 3  (+15 to +180 days) | 1,815.66 | 2,751 | 1.52 | 1.48 (1.40 – 1.57) |
|  |  |  |  |  |  |
| Men >65 years | Control period  (-180 to -15 days) | 4,794.34 | 6,267 | 1.31 | Reference |
|  | Risk period 1  (+15 to +30 days) | 462.11 | 1,346 | 2.91 | 2.23 (2.10 – 2.36) |
|  | Risk period 2  (+15 to +90 days) | 2,195.00 | 4,318 | 1.97 | 1.51 (1.45 – 1.56) |
|  | Risk period 3  (+15 to +180 days) | 4,794.34 | 7,650 | 1.60 | 1.22 (1.18 – 1.26) |

The time-adjusted 2 analyses include a shortened 180-day observation period.

*Time period: time period before (i.e. control period) or after (i.e. risk period 1, risk period 2, risk period 3) antibiotic exposure (in days).

**Person-time: total time contributed to each time period (in years).

***Per PY: per person-year.

IRR indicates incidence rate ratio; CI, confidence interval.

| **Time period*** | **Person-time (years)**** | **UTI events (n)** | **IR (per PY)***** | **IRR (95% CI)** |
| --- | --- | --- | --- | --- |
| Control period (-365 to -15 days) | 422,270.50 | 458,674 | 1.09 | Reference |
| Risk period 1 (+15 to +30 days) | 19,248.80 | 36,427 | 1.89 | 1.74 (1.72 – 1.76) |
| Risk period 2 (+15 to +90 days) | 91,431.80 | 129,299 | 1.41 | 1.30 (1.29 – 1.31) |
| Risk period 3 (+15 to +180 days) | 199,706.30 | 248,533 | 1.24 | 1.15 (1.14 – 1.15) |
| Risk period 4 (+15 to +365 days) | 422,270.50 | 471,628 | 1.12 | 1.03 (1.02 – 1.03) |

**Table 14. Person-time, urinary tract infection events, and incidence rate ratios for the censoring analysis**

The censoring analyses exclude observation periods with death or deregistration within 365 days after oral antibiotic exposure

*Time period: time period before (i.e. control period) or after (i.e. risk period 1, risk period 2, risk period 3, risk period 4) antibiotic exposure (in days).

**Person-time: total time contributed to each time period (in years).

***Per PY: per person-year.

IRR indicates incidence rate ratio; CI, confidence interval.

**Table 15.** **Sex-and age-stratified incidence rate ratios for the censoring analysis**

| **Subgroup** | **Time period*** | **Person-time (years)**** | **UTI events (n)** | **IR (per PY)***** | **IRR (95% CI)** |
| --- | --- | --- | --- | --- | --- |
| Women ≤50 years | Control period  (-365 to -15 days) | 105,768.00 | 76,524 | 0.72 | Reference |
|  | Risk period 1  (+15 to +30 days) | 4,821.33 | 6,886 | 1.43 | 1.97 (1.93 – 2.02) |
|  | Risk period 2  (+15 to +90 days) | 22,901.33 | 23,690 | 1.03 | 1.43 (1.41 – 1.45) |
|  | Risk period 3  (+15 to +180 days) | 50,021.33 | 45,775 | 0.92 | 1.27 (1.25 – 1.28) |
|  | Risk period 4  (+15 to +365 days) | 105,768.00 | 87,342 | 0.83 | 1.14 (1.13 – 1.15) |
|  |  |  |  |  |  |
| Women >50 years | Control period  (-365 to -15 days) | 244,385.37 | 322,490 | 1.32 | Reference |
|  | Risk period 1  (+15 to +30 days) | 11,140.07 | 22,326 | 2.00 | 1.52 (1.50 – 1.54) |
|  | Risk period 2  (+15 to +90 days) | 52,915.35 | 83,722 | 1.58 | 1.20 (1.19 – 1.21) |
|  | Risk period 3  (+15 to +180 days) | 115,578.27 | 164,565 | 1.42 | 1.08 (1.07 – 1.09) |
|  | Risk period 4  (+15 to +365 days) | 244,385.37 | 318,721 | 1.30 | 0.99 (0.98 – 0.99) |
|  |  |  |  |  |  |
| Men ≤65 years | Control period  (-365 to -15 days) | 21,118.62 | 13,294 | 0.63 | Reference |
|  | Risk period 1  (+15 to +30 days) | 962.67 | 2,102 | 2.18 | 3.47 (3.31 – 3.63) |
|  | Risk period 2  (+15 to +90 days) | 4,572.69 | 5,689 | 1.24 | 1.98 (1.92 – 2.04) |
|  | Risk period 3  (+15 to +180 days) | 9,987.72 | 9,639 | 0.97 | 1.53 (1.49 – 1.57) |
|  | Risk period 4  (+15 to +365 days) | 21,118.62 | 16,549 | 0.78 | 1.25 (1.22 – 1.27) |
|  |  |  |  |  |  |
| Men >65 years | Control period  (-365 to -15 days) | 50,998.55 | 46,366 | 0.91 | Reference |
|  | Risk period 1  (+15 to +30 days) | 2,324.72 | 5,113 | 2.20 | 2.42 (2.35 – 2.49) |
|  | Risk period 2  (+15 to +90 days) | 11,042.42 | 16,198 | 1.47 | 1.61 (1.58 – 1.64) |
|  | Risk period 3  (+15 to +180 days) | 24,118.97 | 28,554 | 1.18 | 1.30 (1.28 – 1.32) |
|  | Risk period 4  (+15 to +365 days) | 50,998.55 | 49,016 | 0.96 | 1.06 (1.04 – 1.07) |

The censoring analyses exclude observation periods with death or deregistration within 365 days after oral antibiotic exposure

*Time period: time period before (i.e. control period) or after (i.e. risk period 1, risk period 2, risk period 3, risk period 4) antibiotic exposure (in days).

**Person-time: total time contributed to each time period (in years).

***Per PY: per person-year.

IRR indicates incidence rate ratio; CI, confidence interval.

**Table 16.** **Antibiotic group stratified incidence rate ratios for the censoring analysis**

| **Subgroup** | **Time period*** | **Person-time (years)**** | **UTI events (n)** | **IR (per PY)***** | **IRR (95% CI)** |
| --- | --- | --- | --- | --- | --- |
| β-lactams | Control period  (-365 to -15 days) | 78,923.83 | 72,583 | 0.92 | Reference |
|  | Risk period 1  (+15 to +30 days) | 3,597.67 | 7,467 | 1.80 | 1.96 (1.91 – 2.01) |
|  | Risk period 2  (+15 to +90 days) | 17,088.92 | 22,085 | 1.29 | 1.41 (1.38 – 1.43) |
|  | Risk period 3  (+15 to +180 days) | 37,325.80 | 41,663 | 1.12 | 1.21 (1.20 – 1.23) |
|  | Risk period 4  (+15 to +365 days) | 78,923.83 | 78,910 | 1.00 | 1.09 (1.08 – 1.10) |
|  |  |  |  |  |  |
| Macrolides | Control period  (-365 to -15 days) | 23,727.70 | 21,746 | 0.92 | Reference |
|  | Risk period 1  (+15 to +30 days) | 1,081.60 | 1,110 | 1.03 | 1.12 (1.05 – 1.19) |
|  | Risk period 2  (+15 to +90 days) | 5,137.62 | 5,237 | 1.02 | 1.11 (1.08 – 1.15) |
|  | Risk period 3  (+15 to +180 days) | 11,221.65 | 11,393 | 1.02 | 1.11 (1.08 – 1.13) |
|  | Risk period 4  (+15 to +365 days) | 23,727.70 | 23,936 | 1.01 | 1.10 (1.08 – 1.12) |
|  |  |  |  |  |  |
| Other antibacterials**** | Control period  (-365 to -15 days) | 230,511.62 | 271,320 | 1.18 | Reference |
|  | Risk period 1  (+15 to +30 days) | 10,507.65 | 21,561 | 2.05 | 1.74 (1.72 – 1.77) |
|  | Risk period 2  (+15 to +90 days) | 49,911.35 | 76,165 | 1.53 | 1.30 (1.29 – 1.31) |
|  | Risk period 3  (+15 to +180 days) | 109,016.89 | 145,456 | 1.33 | 1.13 (1.13 – 1.14) |
|  | Risk period 4  (+15 to +365 days) | 230,511.62 | 274,332 | 1.19 | 1.01 (1.01 – 1.02) |
|  |  |  |  |  |  |
| Quinolones | Control period  (-365 to -15 days) | 48,626.83 | 46,079 | 0.95 | Reference |
|  | Risk period 1  (+15 to +30 days) | 2,216.61 | 4,614 | 2.08 | 2.20 (2.13 – 2.26) |
|  | Risk period 2  (+15 to +90 days) | 10,528.89 | 14,805 | 1.41 | 1.48 (1.46 – 1.51) |
|  | Risk period 3  (+15 to +180 days) | 22,997.31 | 27,648 | 1.20 | 1.27 (1.25 – 1.29) |
|  | Risk period 4  (+15 to +365 days) | 48,626.83 | 50,392 | 1.04 | 1.09 (1.08 – 1.11) |
|  |  |  |  |  |  |
| Sulphonamides | Control period  (-365 to -15 days) | 28,762.30 | 47,201 | 1.64 | Reference |
|  | Risk period 1  (+15 to +30 days) | 1,311.10 | 2,985 | 2.28 | 1.39 (1.34 – 1.44) |
|  | Risk period 2  (+15 to +90 days) | 6,277.73 | 11,435 | 1.84 | 1.12 (1.10 – 1.14) |
|  | Risk period 3  (+15 to +180 days) | 13,602.68 | 22,386 | 1.65 | 1.00 (0.99 – 1.02) |
|  | Risk period 4  (+15 to +365 days) | 28,762.30 | 42,074 | 1.46 | 0.89 (0.88 – 0.90) |
|  |  |  |  |  |  |
| Tetracyclines | Control period  (-365 to -15 days) | 18,002.14 | 15,369 | 0.85 | Reference |
|  | Risk period 1  (+15 to +30 days) | 820.61 | 801 | 0.98 | 1.14 (1.06 – 1.23) |
|  | Risk period 2  (+15 to +90 days) | 3,897.90 | 3,686 | 0.95 | 1.11 (1.07 – 1.15) |
|  | Risk period 3  (+15 to +180 days) | 8,513.83 | 8,164 | 0.96 | 1.12 (1.09 – 1.15) |
|  | Risk period 4  (+15 to +365 days) | 18,002.14 | 17,284 | 0.96 | 1.13 (1.10 – 1.15) |

The censoring analyses exclude observation periods with death or deregistration within 365 days after oral antibiotic exposure

*Time period: time period before (i.e. control period) or after (i.e. risk period 1, risk period 2, risk period 3, risk period 4) antibiotic exposure (in days).

**Person-time: total time contributed to each time period (in years).

***Per PY: per person-year.

****Other antibacterials: antibiotics included in the ATC subgroup J01XX ‘’other antibacterials’’.

IRR indicates incidence rate ratio; CI, confidence interval.

**Table 17. Sex-, age-, and β-lactam stratified incidence rate ratios for the censoring analysis**

| **Subgroup** | **Time period*** | **Person-time (years)**** | **UTI events (n)** | **IR (per PY)***** | **IRR (95% CI)** |
| --- | --- | --- | --- | --- | --- |
| Women ≤50 years | Control period  (-365 to -15 days) | 20,596.81 | 16,214 | 0.79 | Reference |
|  | Risk period 1  (+15 to +30 days) | 938.89 | 1,202 | 1.28 | 1.63 (1.53 – 1.72) |
|  | Risk period 2  (+15 to +90 days) | 4,459.71 | 4,449 | 1.00 | 1.27 (1.23 – 1.31) |
|  | Risk period 3  (+15 to +180 days) | 9,740.94 | 8,826 | 0.91 | 1.15 (1.12 – 1.18) |
|  | Risk period 4  (+15 to +365 days) | 20,596.81 | 17,382 | 0.84 | 1.07 (1.05 – 1.10) |
|  |  |  |  |  |  |
| Women >50 years | Control period  (-365 to -15 days) | 38,649.88 | 41,271 | 1.07 | Reference |
|  | Risk period 1  (+15 to +30 days) | 1,761.82 | 3,395 | 1.93 | 1.81 (1.74 – 1.87) |
|  | Risk period 2  (+15 to +90 days) | 8,368.64 | 11,733 | 1.40 | 1.31 (1.29 – 1.34) |
|  | Risk period 3  (+15 to +180 days) | 18,278.86 | 22,470 | 1.23 | 1.15 (1.13 – 1.17) |
|  | Risk period 4  (+15 to +365 days) | 38,649.88 | 43,359 | 1.12 | 1.05 (1.04 – 1.07) |
|  |  |  |  |  |  |
| Men ≤65 years | Control period  (-365 to -15 days) | 5,708.26 | 3,706 | 0.65 | Reference |
|  | Risk period 1  (+15 to +30 days) | 260.21 | 530 | 2.04 | 3.14 (2.86 – 3.44) |
|  | Risk period 2  (+15 to +90 days) | 1,235.98 | 1,604 | 1.30 | 2.00 (1.89 – 2.12) |
|  | Risk period 3  (+15 to +180 days) | 2,699.63 | 2,748 | 1.02 | 1.57 (1.49 – 1.65) |
|  | Risk period 4  (+15 to +365 days) | 5,708.26 | 4,931 | 0.86 | 1.33 (1.28 – 1.39) |
|  |  |  |  |  |  |
| Men >65 years | Control period  (-365 to -15 days) | 13,968.89 | 11,392 | 0.82 | Reference |
|  | Risk period 1  (+15 to +30 days) | 636.76 | 1,340 | 2.10 | 2.58 (2.44 – 2.73) |
|  | Risk period 2  (+15 to +90 days) | 3,024.60 | 4,299 | 1.42 | 1.74 (1.68 – 1.81) |
|  | Risk period 3  (+15 to +180 days) | 6,606.37 | 7,619 | 1.15 | 1.41 (1.37 – 1.46) |
|  | Risk period 4  (+15 to +365 days) | 13,968.89 | 13,238 | 0.95 | 1.16 (1.13 – 1.19) |

The censoring analyses exclude observation periods with death or deregistration within 365 days after oral antibiotic exposure

*Time period: time period before (i.e. control period) or after (i.e. risk period 1, risk period 2, risk period 3, risk period 4) antibiotic exposure (in days).

**Person-time: total time contributed to each time period (in years).

***Per PY: per person-year.

IRR indicates incidence rate ratio; CI, confidence interval.

| **Time period*** | **Person-time (years)**** | **UTI events (n)** | **IR (per PY)***** | **IRR (95% CI)** |
| --- | --- | --- | --- | --- |
| Control period (-365 to -15 days) | 48,457.70 | 25,709 | 0.53 | Reference |
| Risk period 1 (+15 to +30 days) | 2,208.90 | 3,912 | 1.77 | 3.34 (3.23 – 3.45) |
| Risk period 2 (+15 to +90 days) | 10,492.27 | 10,694 | 1.02 | 1.92 (1.88 – 1.97) |
| Risk period 3 (+15 to +180 days) | 22,917.32 | 18,120 | 0.79 | 1.49 (1.46 – 1.52) |
| Risk period 4 (+15 to +365 days) | 48,457.70 | 31,495 | 0.65 | 1.23 (1.21 – 1.25) |

**Table 18. Person-time, urinary tract infection events, and incidence rate ratios for the random antibiotic analysis**

The random antibiotic analyses include a single oral antibiotic exposure per individual; if multiple exposures occurred, one was randomly selected.

*Time period: time period before (i.e. control period) or after (i.e. risk period 1, risk period 2, risk period 3, risk period 4) antibiotic exposure (in days).

**Person-time: total time contributed to each time period (in years).

***Per PY: per person-year.

IRR indicates incidence rate ratio; CI, confidence interval.

**Table 19. Sex-and age-stratified incidence rate ratios for the random antibiotic analysis**

| **Subgroup** | **Time period*** | **Person-time (years)**** | **UTI events (n)** | **IR (per PY)***** | **IRR (95% CI)** |
| --- | --- | --- | --- | --- | --- |
| Women ≤50 years | Control period  (-365 to -15 days) | 16,786.50 | 7,321 | 0.44 | Reference |
|  | Risk period 1  (+15 to +30 days) | 765.20 | 989 | 1.29 | 2.96 (2.77 – 3.17) |
|  | Risk period 2  (+15 to +90 days) | 3,634.68 | 2,852 | 0.79 | 1.80 (1.72 – 1.88) |
|  | Risk period 3  (+15 to +180 days) | 7,938.91 | 4,988 | 0.63 | 1.44 (1.39 – 1.49) |
|  | Risk period 4  (+15 to +365 days) | 16,786.50 | 9,135 | 0.54 | 1.25 (1.21 – 1.29) |
|  |  |  |  |  |  |
| Women >50 years | Control period  (-365 to -15 days) | 21,967.17 | 14,230 | 0.65 | Reference |
|  | Risk period 1  (+15 to +30 days) | 1,001.35 | 1,819 | 1.82 | 2.80 (2.67 – 2.94) |
|  | Risk period 2  (+15 to +90 days) | 4,756.42 | 5,206 | 1.10 | 1.69 (1.64 – 1.74) |
|  | Risk period 3  (+15 to +180 days) | 10,389.03 | 9,114 | 0.88 | 1.35 (1.32 – 1.39) |
|  | Risk period 4  (+15 to +365 days) | 21,967.17 | 16,175 | 0.74 | 1.14 (1.11 – 1.16) |
|  |  |  |  |  |  |
| Men ≤65 years | Control period  (-365 to -15 days) | 3,894.87 | 1,184 | 0.30 | Reference |
|  | Risk period 1  (+15 to +30 days) | 177.54 | 440 | 2.49 | 8.15 (7.31 – 9.10) |
|  | Risk period 2  (+15 to +90 days) | 843.34 | 922 | 1.09 | 3.60 (3.30 – 3.92) |
|  | Risk period 3  (+15 to +180 days) | 1,842.02 | 1,361 | 0.74 | 2.43 (2.25 – 2.63) |
|  | Risk period 4  (+15 to +365 days) | 3,894.87 | 2,062 | 0.53 | 1.74 (1.62 – 1.87) |
|  |  |  |  |  |  |
| Men >65 years | Control period  (-365 to -15 days) | 5,809.16 | 2,974 | 0.51 | Reference |
|  | Risk period 1  (+15 to +30 days) | 264.81 | 664 | 2.51 | 4.90 (4.50 – 5.32) |
|  | Risk period 2  (+15 to +90 days) | 1,257.82 | 1,714 | 1.36 | 2.66 (2.51 – 2.82) |
|  | Risk period 3  (+15 to +180 days) | 2,747.35 | 2,657 | 0.97 | 1.89 (1.80 – 2.00) |
|  | Risk period 4  (+15 to +365 days) | 5,809.16 | 4,123 | 0.71 | 1.39 (1.32 – 1.45) |

The random antibiotic analyses include a single oral antibiotic exposure per individual; if multiple exposures occurred, one was randomly selected.

*Time period: time period before (i.e. control period) or after (i.e. risk period 1, risk period 2, risk period 3, risk period 4) antibiotic exposure (in days).

**Person-time: total time contributed to each time period (in years).

***Per PY: per person-year.

IRR indicates incidence rate ratio; CI, confidence interval.

| **Subgroup** | **Time period*** | **Person-time (years)**** | **UTI events (n)** | **IR (per PY)***** | **IRR (95% CI)** |
| --- | --- | --- | --- | --- | --- |
| β-lactams | Control period  (-365 to -15 days) | 9,447.45 | 5,765 | 0.61 | Reference |
|  | Risk period 1  (+15 to +30 days) | 430.65 | 709 | 1.65 | 2.70 (2.50 – 2.92) |
|  | Risk period 2  (+15 to +90 days) | 2,045.60 | 2,119 | 1.04 | 1.70 (1.62 – 1.78) |
|  | Risk period 3  (+15 to +180 days) | 4,468.03 | 3,779 | 0.85 | 1.39 (1.33 – 1.44) |
|  | Risk period 4  (+15 to +365 days) | 9,447.45 | 6,848 | 0.72 | 1.19 (1.15 – 1.23) |
|  |  |  |  |  |  |
| Macrolides | Control period  (-365 to -15 days) | 2,016.15 | 1,204 | 0.60 | Reference |
|  | Risk period 1  (+15 to +30 days) | 91.90 | 68 | 0.74 | 1.24 (0.97 – 1.58) |
|  | Risk period 2  (+15 to +90 days) | 436.55 | 312 | 0.71 | 1.20 (1.06 – 1.36) |
|  | Risk period 3  (+15 to +180 days) | 953.51 | 729 | 0.77 | 1.28 (1.17 – 1.40) |
|  | Risk period 4  (+15 to +365 days) | 2,016.15 | 1,556 | 0.77 | 1.29 (1.20 – 1.39) |
|  |  |  |  |  |  |
| Other antibacterials**** | Control period  (-365 to -15 days) | 27,008.50 | 13,035 | 0.48 | Reference |
|  | Risk period 1  (+15 to +30 days) | 1,231.16 | 2,261 | 1.84 | 3.81 (3.64 – 3.98) |
|  | Risk period 2  (+15 to +90 days) | 5,848.00 | 5,921 | 1.01 | 2.10 (2.03 – 2.16) |
|  | Risk period 3  (+15 to +180 days) | 12,773.25 | 9,739 | 0.76 | 1.58 (1.54 – 1.62) |
|  | Risk period 4  (+15 to +365 days) | 27,009.50 | 16,407 | 0.61 | 1.26 (1.23 – 1.29) |
|  |  |  |  |  |  |
| Quinolones | Control period  (-365 to -15 days) | 6,199.32 | 2,720 | 0.44 | Reference |
|  | Risk period 1  (+15 to +30 days) | 282.59 | 644 | 2.28 | 5.19 (4.77 – 5.66) |
|  | Risk period 2  (+15 to +90 days) | 1,342.30 | 1,520 | 1.13 | 2.58 (2.42 – 2.75) |
|  | Risk period 3  (+15 to +180 days) | 2,931.87 | 2,376 | 0.81 | 1.85 (1.75 – 1.95) |
|  | Risk period 4  (+15 to +365 days) | 6,199.32 | 3,782 | 0.61 | 1.39 (1.32 – 1.46) |
|  |  |  |  |  |  |
| Sulphonamides | Control period  (-365 to -15 days) | 1,937.35 | 1,799 | 0.93 | Reference |
|  | Risk period 1  (+15 to +30 days) | 88.31 | 158 | 1.79 | 1.93 (1.64 – 2.27) |
|  | Risk period 2  (+15 to +90 days) | 419.48 | 522 | 1.24 | 1.34 (1.22 – 1.48) |
|  | Risk period 3  (+15 to +180 days) | 916.24 | 892 | 0.97 | 1.05 (0.97 – 1.14) |
|  | Risk period 4  (+15 to +365 days) | 1,937.35 | 1,572 | 0.81 | 0.87 (0.82 – 0.94) |
|  |  |  |  |  |  |
| Tetracyclines | Control period  (-365 to -15 days) | 1,765.33 | 1,131 | 0.64 | Reference |
|  | Risk period 1  (+15 to +30 days) | 80.47 | 68 | 0.85 | 1.32 (1.03 – 1.69) |
|  | Risk period 2  (+15 to +90 days) | 382.24 | 286 | 0.75 | 1.17 (1.03 – 1.33) |
|  | Risk period 3  (+15 to +180 days) | 834.89 | 577 | 0.69 | 1.08 (0.98 – 1.20) |
|  | Risk period 4  (+15 to +365 days) | 1,765.33 | 1,274 | 0.72 | 1.13 (1.04 – 1.22) |

**Table 20.** **Antibiotic group stratified incidence rate ratios for the random antibiotic analysis**

The random antibiotic analyses include a single oral antibiotic exposure per individual; if multiple exposures occurred, one was randomly selected.

*Time period: time period before (i.e. control period) or after (i.e. risk period 1, risk period 2, risk period 3, risk period 4) antibiotic exposure (in days).

**Person-time: total time contributed to each time period (in years).

***Per PY: per person-year.

****Other antibacterials: antibiotics included in the ATC subgroup J01XX ‘’other antibacterials’’

IRR indicates incidence rate ratio; CI, confidence interval.

**Table 21. Sex-, age-, and β-lactam stratified incidence rate ratios for the random antibiotic analysis**

| **Subgroup** | **Time period*** | **Person-time (years)**** | **UTI events (n)** | **IR (per PY)***** | **IRR (95% CI)** |
| --- | --- | --- | --- | --- | --- |
| Women ≤50 years | Control period  (-365 to -15 days) | 3,035.75 | 1,851 | 0.61 | Reference |
|  | Risk period 1  (+15 to +30 days) | 138.38 | 135 | 0.98 | 1.60 (1.34 – 1.91) |
|  | Risk period 2  (+15 to +90 days) | 657.31 | 508 | 0.77 | 1.27 (1.15 – 1.40) |
|  | Risk period 3  (+15 to +180 days) | 1,435.71 | 979 | 0.68 | 1.12 (1.04 – 1.21) |
|  | Risk period 4  (+15 to +365 days) | 3,035.75 | 1,943 | 0.64 | 1.05 (0.99 – 1.12) |
|  |  |  |  |  |  |
| Women >50 years | Control period  (-365 to -15 days) | 3,855.47 | 2,612 | 0.68 | Reference |
|  | Risk period 1  (+15 to +30 days) | 175.75 | 314 | 1.79 | 2.64 (2.35 – 2.97) |
|  | Risk period 2  (+15 to +90 days) | 834.80 | 902 | 1.08 | 1.60 (1.48 – 1.72) |
|  | Risk period 3  (+15 to +180 days) | 1,823.39 | 1,662 | 0.91 | 1.35 (1.27 – 1.43) |
|  | Risk period 4  (+15 to +365 days) | 3,855.47 | 3,038 | 0.79 | 1.16 (1.10 – 1.23) |
|  |  |  |  |  |  |
| Men ≤65 years | Control period  (-365 to -15 days) | 955.22 | 397 | 0.42 | Reference |
|  | Risk period 1  (+15 to +30 days) | 43.54 | 95 | 2.18 | 5.25 (4.20 – 6.57) |
|  | Risk period 2  (+15 to +90 days) | 206.83 | 246 | 1.19 | 2.86 (2.44 – 3.36) |
|  | Risk period 3  (+15 to +180 days) | 451.76 | 379 | 0.84 | 2.02 (1.75 – 2.32) |
|  | Risk period 4  (+15 to +365 days) | 955.22 | 641 | 0.67 | 1.62 (1.43 – 1.83) |
|  |  |  |  |  |  |
| Men >65 years | Control period  (-365 to -15 days) | 1,601.00 | 905 | 0.57 | Reference |
|  | Risk period 1  (+15 to +30 days) | 72.98 | 165 | 2.26 | 4.00 (3.39 – 4.72) |
|  | Risk period 2  (+15 to +90 days) | 346.66 | 463 | 1.34 | 2.36 (2.11 – 2.64) |
|  | Risk period 3  (+15 to +180 days) | 757.17 | 759 | 1.00 | 1.77 (1.61 – 1.95) |
|  | Risk period 4  (+15 to +365 days) | 1,601.00 | 1,226 | 0.77 | 1.36 (1.24 – 1.48) |

The random antibiotic analyses include a single oral antibiotic exposure per individual; if multiple exposures occurred, one was randomly selected.

*Time period: time period before (i.e. control period) or after (i.e. risk period 1, risk period 2, risk period 3, risk period 4) antibiotic exposure (in days).

**Person-time: total time contributed to each time period (in years).

***Per PY: per person-year.

IRR indicates incidence rate ratio; CI, confidence interval.

**Text 1. English translation of the Dutch national guideline text on watchful waiting for urinary tract infections**

**Clinical question**

Is a watchful waiting approach, with analgesics if needed, (compared with antibiotics) recommended in healthy, non-pregnant women with a urinary tract infection in primary care? (see PICO table).

**PICO**

| **Component** | **Description** |
| --- | --- |
| Patients | Healthy, non-pregnant women with a urinary tract infection in primary care |
| Intervention | Analgesics or placebo |
| Comparison | Antibiotics |
| Outcomes | (Acceptance – not identified in studies) Clinical course based on symptom resolution, adverse effects / complications, and antibiotic use; recurrent risk |

**Background**

Urinary tract infection is a common reason for visiting the general practitioner, for which antibiotics are frequently prescribed. Antibiotic use may contribute to antibiotic resistance. Antibiotics may not always be necessary, and a watchful waiting approach with analgesics may represent an alternative for certain patient groups.

**Methods**

A systematic literature search for systematic reviews and randomized controlled trials (RCTs) was performed in PubMed in May 2018.

**Results**

**Search results**

A review on the diagnosis and treatment of urinary tract infections in primary care served as the basis for answering the clinical question.^1^ In this review, the comparison between antibiotic prescribing and analgesic treatment for urinary tract infections was included.

After publication of this review, three additional studies on analgesic treatment versus antibiotic prescribing for urinary tract infections were published.^2-5^

**Study characteristics**

Grigoryan included four RCTs (n = 1609)^6-9^ comparing antibiotic prescribing (nitrofurantoin, pivmecillinam, ciprofloxacin) with non-antimicrobial treatment (placebo or ibuprofen) in non-pregnant women with urinary tract infection without signs of tissue invasion in primary care. Absence of symptoms after 4 (7 or 8–10) days was the primary outcome.

**Gagyor 2015^3^:** RCT (n = 494) conducted in 42 German general practices. Female patients aged 18–65 years with typical urinary tract infection symptoms and without risk factors or complications were included and randomly assigned to either fosfomycin (3 g) (n = 246) or ibuprofen (3 × 400 mg) (n = 248). Treatment duration was three days. Patients in the fosfomycin group received a single dose of fosfomycin followed by placebo. Patients were advised to revisit their GP if symptoms persisted or worsened, after which antibiotic treatment could be initiated based on urine culture results obtained during the first visit. Primary outcomes were the number of antibiotic prescriptions by day 28 and symptom burden (dysuria, urinary frequency, lower abdominal pain) on day 7. A follow-up study monitored these women for six months after inclusion, reporting recurrences and complications.

**Kronenberg 2017^4^:** Double-blind RCT (n = 253) conducted in 17 Swiss general practices. Women with uncomplicated UTI were randomized to either diclofenac (NSAID) (n = 133) or norfloxacin antibiotic treatment (n = 120). The primary outcome was absence/reduction of symptoms on day 3. Secondary outcome was antibiotic use up to day 30.

**Vik 2018^5^:** Double-blind RCT (n = 383) conducted in 16 general practices in Norway, Sweden, and Denmark. Non-pregnant women with symptoms of urinary tract infection without signs of tissue invasion were randomized to either ibuprofen three times daily for three days (n = 194) or pivmecillinam three times daily for three days (n = 189). The primary outcome was the proportion of patients feeling improved/cured on day 4. Secondary outcomes included the proportion requiring a second course of antibiotics and cases of pyelonephritis.

**Conclusions**

Compared with a watchful waiting approach, antibiotic prescribing resulted in up to 37% more women being symptom-free after 3–10 days (−6% to 35% on day 3–4 and 12% to 37% on day 7–10). The working group considered these differences between study groups clinically relevant.

A watchful waiting approach reduced the number of antibiotic prescriptions.

Based on the small number of events across studies, a watchful waiting approach may be associated with a slightly increased risk of pyelonephritis (incidence approximately 2%, absolute risk difference compared with treated populations approximately 1.6%).

The risk of recurrent urinary tract infection was similar between watchful waiting and antibiotic prescribing.

Table D8. Comparison of watchful waiting/analgesic treatment and antibiotic prescribing for urinary tract infection with respect to symptom resolution, antibiotic use, complications (pyelonephritis), and recurrence risk

|  |  | **Symptom resolution** | | **Antibiotic use** | | **Complications** | | **Recurrence risk** | |
| --- | --- | --- | --- | --- | --- | --- | --- | --- | --- |
| **RCT** | **N** | **Watchful waiting** | **Antibiotics** | **Watchful waiting** | **Antibiotics** | **Watchful waiting** | **Antibiotics** | **Watchful waiting** | **Antibiotics** |
| Christiaens Belgium  2002 (nitrofurantoin) | 78 | Day 7: 14/33 (42%) | Day 7: 24/34 (70%) | Unknown | 100% | 1/38 (3%) | 0/40 (0%) | Unknown | Unknown |
|  |  |  |  |  |  |  |  |  |  |
| Ferry  Sweden  2007 (pivmecillinam) | 1143 | Day 8–10: 53/212 (25%) | Day 8–10: 132/213 (62%) | Unknown | 100% | 1/288 (0.3%) | 1/855 (0.1%) | Unknown | Unknown |
|  |  |  |  |  |  |  |  |  |  |
| Little  UK  2010 (trimethoprim) | 309 | Unknown | Unknown | 41/53 (77%) | 58/60 (97%) | 0/53 (0%) | 0/60 (0%) | Unknown | Unknown |
|  |  |  |  |  |  |  |  |  |  |
| Bleidorn Germany  2010 (ciprofloxacin) | 79 | Day 4: 21/36 (58%) | Day 4: 17/33 (52%) | 12/36 (33%) | 33/33 (100%) | 0/36 (0%) | 0/33 (0%) | Unknown | Unknown |
|  |  |  |  |  |  |  |  |  |  |
| Gagyor  Germany  2015 (fosfomycin) | 494 | Day 4: 91/234 (39%); Day 7: 163/232 (70%) | Day 4: 129/229 (56%); Day 7: 186/227 (82%) | 75/241 (31%) | 243/243 (100%) | Day 1–28: 5/241 (2%) | Day 1–28: 1/243 (0.4%) | Day 15–28: 14/241 (6%) | Day 15–28: 27/243 (11%) |
|  |  |  |  |  |  |  |  |  |  |
|  |  |  |  |  |  |  |  |  |  |
| Bleidorn Germany  2016 (fosfomycin) | 494 | N/A | N/A | N/A | N/A | Day 28–6 months: 1/122 (1%) | Day 28–6 months: 2/264* (1%) | Day 28–6 months: 24/122 (20%) | Day 28–6 months: 56/264* (21%) |
|  |  |  |  |  |  |  |  |  |  |
| Kronenberg Switzerland  2017 (norfloxacin) | 253 | Day 3: 72/133 (54%); Day 7: 111/133 (83%) | Day 3: 96/120 (80%); Day 7: 115/120 (96%) | 82/133 (62%) | 118/120 (98%) | 6/133 (5%) | 0/120 (0%) | After day 14: 5/133 (4%) | After day 14: 4/120 (3%) |
|  |  |  |  |  |  |  |  |  |  |
| Vik  Norway, Sweden and Denmark  2018 (pivmecillinam) | 383 | Day 4: 70/181 (39%); Day 7: 114/181 (63%) | Day 4: 131/178 (74%); Day 7: 162/178 (91%) | After 4 weeks: 47% | 100% | 7/181 (4%) | 0/178 (0%) | Day 15–28: 10/181 (6%) | Day 15–28: 4/178 (2%) |

*Greater than the original group size because patients from the “watchful waiting” group who ultimately received antibiotics were also included.

**From evidence to recommendation**

**Benefits and harms**

There appears to be a clinically relevant difference in the speed of symptom resolution between watchful waiting/analgesics and antibiotic treatment, favoring antibiotics. No difference appears to exist in recurrence risk. The risk of pyelonephritis may be slightly increased. It is unknown whether a previous episode of pyelonephritis increases the risk of subsequent pyelonephritis under a watchful waiting approach.

**Quality of evidence**

Due to heterogeneity between studies and small sample sizes, the quality of evidence was considered low.

**Patient values and preferences**

For healthy, non-pregnant women with urinary tract infection who prefer to avoid antibiotics, a watchful waiting approach may be offered instead of antibiotics. The balance between symptom duration (individual), risk of complications or adverse effects (individual), and antibiotic resistance (community and individual) will differ between patients.

**Why this recommendation?**

Although symptom duration may be somewhat longer and the risk of pyelonephritis may be slightly increased, avoidance of antibiotics may nevertheless be a valid reason to choose analgesics/watchful waiting.

**Acceptance of delayed antibiotic prescribing**

In a study conducted in 20 general practices in Amsterdam, 51 of 137 women with a urinary tract infection (37%) were willing to delay antibiotic treatment.^10^ After one week, 55% of these patients (28/51) had in fact not used antibiotics.

In an Irish study in which 14 patients were interviewed, 50% indicated that they would be willing to delay antibiotic treatment.^11^ In addition to a desire for antibiotic treatment, symptom relief and reassurance were important reasons for consulting the general practitioner. Willingness to delay antibiotic treatment appeared to depend on factors such as symptom severity, previous attempts at self-management, prior experience with delayed prescriptions, and previous experiences with such approaches.

In a Dutch study, 47% of patients indicated that they wanted antibiotics for pain reduction, whereas only 29% used analgesics.^12^ In this study, patients who were less willing to delay antibiotic treatment presented earlier (mean symptom duration 3.3 days versus 4.4 days among patients willing to delay treatment). In the study by Knottnerus,^10^ patients who believed they had a urinary tract infection, or who had urine dipstick findings suggestive of a urinary tract infection, were less willing to delay antibiotic treatment. Willems additionally showed that 50% of patients were aware that UTIs may follow a self-limiting course. However, this did not influence acceptance of a delayed prescription.

In the same study, increased use of nitrofurantoin was observed, mainly due to an increase in consultations coded with ICPC U02 (frequent urination) from 15 to 20.2 per 1000 person-years and U71 (cystitis) from 78.1 to 93.4 per 1000 person-years between 2007 and 2011. The incidence of cystitis likely did not increase; rather, these findings may reflect a lower threshold for consulting primary care. The authors suggested that this may be related to the fact that, during the later period, antibiotics could also be prescribed based on symptoms alone.

**Conclusion**

Patients wishing to avoid antibiotics will likely respond positively to a watchful waiting approach, although for other healthy, non-pregnant women, watchful waiting may also represent a suitable alternative to antibiotic treatment. The balance between symptom duration (individual), risk of complications (individual), and antibiotic resistance (community and individual) will differ between patients.

**References**

**1** Grigoryan L, Trautner BW, Gupta K. Diagnosis and management of urinary tract infections in the outpatient setting: A review. *JAMA* 2014;312:1677-84.

**2** Gagyor I, Bleidorn J, Kochen MM, et al. Ibuprofen versus fosfomycin for uncomplicated urinary tract infection in women: Randomised controlled trial. *BMJ* 2015;351:h6544.

**3** Bleidorn J, Hummers-Pradier E, Schmiemann G, et al. Recurrent urinary tract infections and complications after symptomatic versus antibiotic treatment: Follow-up of a randomised controlled trial. *Ger Med Sci* 2016;14:Doc01.

**4** Kronenberg A, Butikofer L, Odutayo A, et al. Symptomatic treatment of uncomplicated lower urinary tract infections in the ambulatory setting: Randomised, double blind trial. *BMJ* 2017;359:j4784.

**5** Vik I, Bollestad M, Grude N, et al. Ibuprofen versus pivmecillinam for uncomplicated urinary tract infection in women-a double-blind, randomized non-inferiority trial. *PLoS Med* 2018;15:e1002569.

**6** Christiaens TC, De Meyere M, Verschraegen G, et al. Randomised controlled trial of nitrofurantoin versus placebo in the treatment of uncomplicated urinary tract infection in adult women. *Br J Gen Pract* 2002;52:729-34.

**7** Ferry SA, Holm SE, Stenlund H, et al. Clinical and bacteriological outcome of different doses and duration of pivmecillinam compared with placebo therapy of uncomplicated lower urinary tract infection in women: The LUTIW project. *Scand J Prim Health Care* 2007;25:49-57.

**8** Little P, Moore MV, Turner S, et al. Effectiveness of five different approaches in management of urinary tract infection: Randomised controlled trial. *BMJ* 2010;340:c199.

**9** Bleidorn J, Gagyor I, Kochen MM, et al. Symptomatic treatment (ibuprofen) or antibiotics (ciprofloxacin) for uncomplicated urinary tract infection?--results of a randomized controlled pilot trial. *BMC Med* 2010;8:30.

**10** Knottnerus BJ, Geerlings SE, Moll van Charante EP, et al. Women with symptoms of uncomplicated urinary tract infection are often willing to delay antibiotic treatment: A prospective cohort study. *BMC Fam Pract* 2013;14:71.

**11** Duane S, Beatty P, Murphy AW, et al. Exploring experiences of delayed prescribing and symptomatic treatment for urinary tract infections among general practitioners and patients in ambulatory care: A qualitative study. *Antibiotics (Basel)* 2016;5:pii: E27.

**12** Willems CS, Van den Broek D'Obrenan J, Numans ME, et al. Cystitis: Antibiotic prescribing, consultation, attitudes and opinions. *Fam Pract* 2014;31:149-55.
