# Supplementary material for: Oral antibiotic exposure and urinary tract infection risk in adults: a self-controlled case series study
Source: eClinicalMedicine. 2026 Jul 14;97:104060. doi: 10.1016/j.eclinm.2026.104060 (PMC13382391; doi:10.1016/j.eclinm.2026.104060)

**Protocol for (medical) scientific research using existing (patient) data (non-WMO) at the Leiden University Medical Center (LUMC)**

1. **Information about the researcher(s) and funder(s)**

| **1.1**  **Research title** | The association between antibiotic use and risk of urinary tract infection in adults |
| --- | --- |
| **1.2**  **Researcher(s)** | Mirjam Deelen  Master student Health Sciences  VU Amsterdam  Dr. Mirte Boelens  Epidemiologist  LUMC; department of Public Health and Primary Care  Dr. Hanneke Borgdorff  General practitioner & researcher  LUMC; department of Public Health and Primary Care  Dr. Merel Lambregts  Internal medicine and infectious diseases physician & researcher  Leiden University Center of Infectious Diseases  Dr. Martijn Sijbom  General practitioner & researcher  LUMC; department of Public Health and Primary Care  Dr. Cees van Nieuwkoop  Internal medicine and infectious diseases physician & researcher  Haga Hospital; department of internal medicine  LUMC; department of Public Health and Primary Care |
| **1.3**  **Principal investigator(s) / sponsor(s)** | LUMC; department of Public Health and Primary Care |
| **1.4**  **Protocol date** | December 18th, 2024 |

1. **Study objective(s)**

| **2.1**  **Research question/objective(s) of the study** | The aim is to assess the association between antibiotic use and risk of subsequent urinary tract infections in adults.  ***Background***  Urinary tract infections (UTIs) encompass a spectrum of infectious syndromes that affect the urinary tract anywhere from the urethra to the kidneys and are one of the most common infections, particularly in primary care (Al Hawati, Blair & Larnard). In 2023, the UTI incidence in primary care was 20 cases per 1.000 registered men and 117 cases per 1.000 registered women (Nivel, 2024). Not only is the UTI incidence higher in women than men, it also increases with age (Ahmet et al., 2018).  Women are particularly susceptible due to the close proximity of the rectum to the urethra as well as a relatively short urethral length (Li & Leslie, 2023). It is reported that 50-60% of women have at least one UTI in their lifetime (Al Hawati, Blair & Larnard, 2023). Most UTI are caused by bacteria from the gut  (Worby et al. 2022).  Most patients with UTIs can be treated relatively simple with oral antibiotics, although treatment failure occurs. Around 20-30% of women with an initial UTI will experience a recurrent UTI within 3-4 months of initial infection (Foxman, 2003). Furthermore, some UTIs can become complicated through a pyelonephritis, prostatitis, and urosepsis, which are associated with morbidity and mortality (Sabih & Leslie, 2023).  A healthy vaginal microbiota is low-diverse and dominated by *Lactobacillus crispatus*.  Vaginal lactobacilli have been touted to prevent invasion of uropathogens, for instance by creating an acidic environment. Disruption of the vaginal ecosystem, causing an overgrowth of anaerobic bacteria or yeasts, increases the risk of vaginal infections such as sexually transmitted infections (Stapleton, 2016, Herthelius 1988). Conversely, treatment that restores a Lactobacillus-dominated microbiota is associated with lower risk of recurrent UTIs. Usage of antibiotics can alter the microbial community (Chee, Chew & Than, 2020).  In pre-menopausal women, factors related to sexual behaviour are major risk factors for UTIs. These include: having a new sexual partner, increased frequency of sexual intercourse as well as the use of spermicide-containing contraceptives (Hooton, 2001). Sexual behaviour is less firmly associated with UTIs in post-menopausal women, where age-, hormonal- and disease-related urogynaecology and immune factors play a more prominent role. An increased postvoid residual urine volume, the presence of bladder prolapse and urinary incontinence have been associated with UTIs (Athanasiou et al., 2010). Antibiotic use or treatment of asymptomatic bacteriuria has also been associated with the development of UTIs (Cai et al., 2014, Smith et al. 1997).  To date, only one small-scale study has been conducted on this subject, which indicated a potential correlation between antibiotic use and an elevated risk of urinary tract infections in pre-menopausal women (Smith et al. 1997). To our knowledge, there are no studies whether this also holds for postmenopausal women and men.  In this study, we would like to evaluate whether different antibiotics (either to treat UTI or other infections) are associated with increased risk of UTI in premenopausal women, postmenopausal women and men. If this association can be confirmed, the next would be to study effective preventive measure for an UTI. This would be highly beneficial, as urinary tract infections represent a significant burden for patients, while antibiotic use is also the major contributing factor to the development of antibiotic resistance. |
| --- | --- |

1. **Study design**

| **3.1**  **a. General description of the study design** | This research project will use the data infrastructure Extramural Leiden Academic Network (ELAN).  ELAN provides access to and the use of pseudonymized healthcare data from various cooperating partners such as healthcare data of general practitioners. The study period concedes 2014-2024.  ***Question***  Is antibiotic use associated with increased risk for urinary tract infection in adults?  Further explorations will be done to what extent the increased UTI risk of antibiotic use holds for:   - Different antibiotics or antibiotic classes - Men versus women - Post- versus Premenopausal women - Indication for antibiotic treatment (excluding UTI’s)   A self-controlled cases series (SCCS) design will be employed to establish the risk for developing an UTI within 3 months after antibiotic treatment compared to patients with an UTI who did not receive an antibiotic treatment. With the SCCS design, patients will be their own control.  The primary endpoint is risk of a UTI within 3 months after initiation of antibiotic treatment computed as an odds ratio.  An antibiotic treatment is defined as the exposure. An UTI between 2 weeks and 3 months after antibiotic treatment is defined as an event. The first 2 weeks after start of the treatment, patients are still somewhat protected by the antibiotic use against a new infection. And if a new antibiotic treatment is needed within 2 weeks after start of treatment, this is considered as treatment failure of the initial treatment.  Descriptive analysis will be performed for exposures, outcomes and covariables for the whole population of analysis and stratified by antibiotic use or not.  Multivariable logistic regression analysis will be performed to assess whether there is an association of antibiotic use with developing a UTI within 3 months after initiation of treatment. First a crude model will be used followed by confounder adjusted models. Confounders are season variability (Winter, spring, summer and autumn) and risk factors for UTI and selected based on theory or literature (Tj VanderWeele 2019). Risk factors are a history of an UTI, urine incontinence, urolithiasis, pregnancy, urine catheter, anatomical / functional abnormality urinary tract, immunocompromising comorbidities such as HIV/AIDS ,Type 2 Diabetes Mellitus.  **Subgroup analysis**  Subsequently, subgroup analysis will be performed to examine whether the association differs upon specific type of antibiotic treatment. For each subgroup, we will calculate an odds ratio for an UTI after antibiotic treatment. Subgroups are antibiotic(group)s, gender and age, type of infection and social determinants .  *Most used antibiotic(group)s in primary care*:   - Amoxicillin - Amoxicillin/clavulanic acid - Flucloxacillin - Tetracyclines - Macrolides - Fluoroquinolones - Trimethoprim - Co-trimoxazole - Fosfomycin - Nitrofurantoin   *Type of infection*   - All infections - UTI - non-UTI (all other infections)   *Gender and age*   - Men - Premenopausal women, defined as < 50 years of age - Postmenopausal women, defined as ≥ 50 years of age   *Social determinants*   - Socio-economic status (SES) - Migrant background |
| --- | --- |
| **3.2**  **a. When will the study be conducted?**  **b. What is the intended end date of the study?** | The research will be performed as part of a MSc. Internship (M. Deelen) in the period between February 2025 and June 2025.  Month 1 – Literature research, writing the introduction, first part of data preparation.  Month 2 – Writing the introduction and methodology, second part of data preparation.  Month 3 – Completing the introduction, methodology and data preparation, start of data analysis.  Month 4 – Completing the data analysis and writing the results.  Month 5 – Writing the report, focusing on the discussion.  The end date is July 2025. |
| **3.3**  **Does the study involve exclusively existing (patient) data (retrospective) and not data yet to be generated (prospective)?** | This study includes already existing data only, and is thus retrospective (see also 3.1). |
| **3.4**  **What type of data will be used for the research?** | Pseudonymized routine care patient data from GP practices participating in ELAN. |
| **3.5**  **If information from patient records is used: describe what type of information is involved.** | The dataset from ELAN contains data over a period of 10 years and includes:   - Number of all included patients in ELAN per year - All patients with an UTI   - ICPC code U70, U71, including subcodes   - Date of registration with GP office - Antibiotic prescriptions ATC J01 for all patients with an UTI   - Corresponding medical diagnosis with the antibiotic prescription by ICPC code   - Details on the antibiotic prescription (name of antibiotic, dose and duration) - Characteristics of included patients   - Year of birth   - Year of death   - Sex   - Referral to urologist for recurrent UTIs   - Risk factors for an UTI     - History of UTI ICPC code U70, U71     - Urine incontinence ICPC code U04     - Urolithiasis ICPC code U95     - Urine catheter ICPC code U28     - Anatomical / functional abnormality urinary tract ICPC code U85     - Type 2 Diabetes Mellitus ICPC code T90.02     - Prostate hypertrophy ICPC codes Y06, Y85     - Medication use for miction problems with ATC codes     - G04BD     - G04C     - Social economic status     - Migrant background |
| **3.6**  **To which time period do the data relate?** | The period between 2014 and 2024 |

1. **Data storage and privacy**

| **4.1**  **a. In the context of this study, will patients/participants be asked for consent to use their (medical) data?**  **b. If no consent is requested: please explain why consent will not be sought.** | In general, it can be stated for the ELAN data warehouse that, in accordance with current guidelines, patients are given the opportunity, through an informed opt-out procedure, to NOT make their pseudonymized and coded data available for scientific research. To inform patients, (digital) posters are hung in the waiting rooms. There are also leaflets available that patients can take with them. The information also appears on the websites of the practices (see supplement A).  Not applicable |
| --- | --- |
| **4.2**  **Have patients/participants previously been asked for consent to use their (medical) data for scientific research?** | See 4.1 |
| **4.3**  **a. Is there (or has there been) a treatment relationship between the researcher(s), or the researcher(s)’ department, and the patients whose records are being reviewed? (see also section 4.8 below)**  **b. Is the treatment relationship still ongoing, or has it since been terminated?** | Yes, two of the researchers (Martijn Sijbom, Hanneke Borgdorff) worked as a general practitioner in the region where the research is done. Next to pseudonymization of patients, details on GP and GP practice is pseudonymized as well.  The principle investigator of this project has stopped working in one of the ELAN data warehouse practices and there is no longer a treatment relation. |
| **4.4**  **Is it likely that patients from the study population have since passed away?** | Yes, this is a possibility. |
| **4.5**  **For how many patients will data be used?** | The study will use data of all patients of 18 year and older in the ELAN data warehouse. |
| **4.6**  **Have patients had the (general) opportunity to object to the (coded/anonymized) use of their (medical) data?** | Yes, within the framework of the ELAN data warehouse, registered patients are informed by participating GPs about the reuse of their medical data for scientific research, with leaflets and posters. Hereby they are offered the opportunity to lodge an objection (informed opt-out procedure). |
| **4.7**  **Is the use of (medical) data for scientific purposes recorded in the medical record of the relevant patient(s)?** | No explicit note is made per patient in the EPD of the general practitioner, because most general practitioner systems are not (yet) designed for this. It is, however, recorded which patients object to reuse their data for research and it is not used for research. |
| **4.8**  **a. By whom will the required data be extracted from the patient records?**  **b. Are the individuals who extract the required data from the patient records authorized to do so on the basis of a treatment relationship with the respective patients?**  **c. If the answer to question 4.8 is no: are the individuals who extract the data from the patient records under the direct supervision of a clinician who, by virtue of a treatment agreement, is authorized to access the records? If so, please state the name and position of the relevant clinician(s).** | The data is extracted from the file by an external party, as described in the governance document of the ELAN data warehouse: <https://www.lumc.nl/sub/4070/att/1914340/ELAN-Gov-okt2019>.  The external party is the Foundation for Information Provision for Care and Research (STIZON), which has entered into a service and processing agreement for this with the general practitioner. STIZON is ISO 27001 and NEN 7510 certified.  No.  Yes, the general practitioners (patients' caregivers) have instructed STIZON (see above) to extract and provide pseudonymized patient data from the general practitioner information system for the ELAN data warehouse. Processor agreements and / or service agreements have been signed for this between GPs and the data supplier. |
| **4.9**  **Will personally identifiable data be made available to the researcher(s)?** | No, conversion to individual natural persons is reasonably prevented. Analyzes are performed in the secure setting of CBS. Before the results can be exported from the CBS environment, the CBS carries out a mandatory output check where the risk of disclosure is checked. Every result from this research must comply with the output guidelines of the CBS (see Appendix B). For example, all tables and similar output must contain at least 10 units (unweighted) as the basis for each cell or data point. In this way it is independently tested and guaranteed that no data traceable to an individual or institution can be used. |
| **4.10**  **If coding takes place: when will coding occur, by whom, and in what manner?** | Coding is done by STIZON and CBS |
| **4.11**  **Has a notification of the intended data processing been submitted via:**  <https://www.albinusnet.nl/weten-en-regelen/juridische-zaken/privacy/meldenverzamelenonderzoeksdata/>**?** | Yes |
| **4.12**  **If applicable: where will the coded data be stored, and who will have access to the coded data?**  **If applicable: where will the uncoded data be stored, and who will have access to the uncoded data?** | The coded data is stored, and a specific research folder assigned to the department disk (I: \) of PHEG. Only the researchers involved (Martijn Sijbom and Mirjam Deelen) and the PHEG data manager have access to this folder.  There is no storage of uncoded data. |
| **4.13**  **What technical and organizational measures have been taken to prevent loss, theft, or unauthorized use of the research data? For example, is a data safe being used?** | The data never physically leaves the research folder of the LUMC or the remote access CBS environment and see 4.12. |
| **4.14**  **By whom is the code key managed?** | STIZON. |
| **4.15**  **Will (research) data be shared with another institution(s) within the Netherlands and/or the EU?** | No. |
| **4.16**  **a. Will (research) data be shared with another institution/organization outside the EU?**  **b. Will the relevant patients be asked for consent to share their personal data with a country outside the EU?** | No.  Not applicable. |
| **4.17**  **How long will the (research) data be retained? If the data will be kept for longer or shorter than the standard retention period of 15 years, please explain why.** | After processing, the research data is returned to ELAN in accordance with the data transfer agreement. The LUMC department PHEG will keep the research data for 15 years. |

1. **Research population**

| **5.1**  **Inclusion criteria** | Adults patients 18 year and older with an acute UTI and registered at an GP office for a least 1 year |
| --- | --- |
| **5.2**  **Exclusion criteria** | Patients younger than 18 years of age.  Patients with antibiotic prophylaxes  UTIs are excluded as an event if   - During pregnancy - within 3 months after giving birth - within 1 year prior to an UTI (wash-our period) |

1. **Statistical analysis**

| **6.1**  **a. Primary outcome**  **b. Secondary outcome** | ***Primary outcome***  What is the association of antibiotic use for any infection with risk for urinary tract infection in adults?   - Odds ratio for an UTI after antibiotic use in the preceding 3 months   - Per infection group, antibiotic for     - All infections     - An UTI     - An infection expect UTI’s   - Per antibiotic (group)   - Per patient group     - Men     - Women < 50 years of age     - Women ≥ 50 years of age   - Social determinants     - SES     - Migrant background   - Combinations of each subgroup   ***Secondary outcome***   - Hazard ratio for an UTI after antibiotic use in the preceding 3 months   - Per infection group, antibiotic for     - All infections     - UTI     - non-UTI   - Per antibiotic group   - Per patient group     - Men     - Women < 50 years of age     - Women ≥ 50 years of age   - Social determinants     - SES     - Migrant background   - Combination of each subgroup - Incidence of an UTI per month |
| --- | --- |
| **6.2**  **a. Statistical analysis primary outcome**    **b. Statistical analysis secondary outcome** | ***Primary outcome***  The Odds ratio for an UTI after antibiotic treatment the preceding 3 months will be calculated through a logistic regression model.  Risk factors and season variability are defined as confounders in this logistic regression model. Risk factors are a history of an UTI, urine incontinence, urolithiasis, pregnancy, urine catheter, anatomical / functional abnormality urinary tract and Type 2 Diabetes Mellitus. An Odds ratio for an UTI will be attributed to each confounder based on a critical literature appraisal.  ***Secondary outcome***  Cox proportional hazard regression models will be used to examine the associations between an UTI after antibiotic treatment the preceding 3 months.  Survival time (in days) was defined as the period between 14 days after the start of the antibiotic treatment and an UTI. The hazard ratio will be adjusted with the previous described confounders (see primary outcome).  The incidence of each UTI will be calculated for each month during 10 years to examine the season variability. |

1. **Appendices and references**

| **7.1**  **Appendix** | **Appendix A (concerning GP-data):**  Patient Brochure (see this link: <https://www.lumc.nl/siteassets/over-het-lumc/partners/elan/elan-folder-nl.pdf>) &  Poster of the ELAN Datawarehouse (see this link: <https://www.lumc.nl/over-het-lumc/partners/elan/>) (including the English version). |
| --- | --- |
| **7.2**  **References** | Ahmed, H., Farewell, D., Jones, H.M., Francis, N.A., Paranjothy, S. & Butler, C.C. (2018). Incidence and antibiotic prescribing for clinically diagnosed urinary tract infection in older adults in UK primary care, 2004-2014. PLoS One, 13(1), e0190521. <https://doi.org/10.1371/journal.pone.0190521>.  Al Hawati, H. Blair, B.M. & Larnard, J. (2023). Urinary Tract Infections: Core Curriculum 2024. American Journal of Kidney Diseases, 83(1), 90-100. <https://doi.org/10.1053/j.ajkd.2023.08.009>.  Athanasiou, S., Antsaklis, A., Betsi, G.I., Sotiropoulou, M. & Falagas, M.E. (2010). Clinical and urodynamic parameters associated with history of urinary tract infections in women: a prospective study. Acta Obstretica et Gynecologica Scandinavica, 86(9), 1130-1135. <https://doi.org/10.1080/00016340701446181>.  Cai, T., Mazzoli, S., Migno, S., Malossini, G., Lanzafame, P., Mereu, L., Tateo, S., Wagenlehner, F.M.E., Pickard, R.S. & Bartoletti, R. (2014). Development and validation of a nomogram predicting recurrence risk in women with symptomatic urinary tract infection. International Journal of Urology, 21(9), 929-934. <https://doi.org/10.1111/iju.12453>.  Chee, W.J.Y., Chew, S.Y. & Than, L.T.L. (2020). Vaginal microbiota and the potential of Lactobacillus derivates in maintaining vaginal health. Microbial Cell Factories, 19, 203. <https://doi.org/10.1186/s12934-020-01464-4>.  Foxman, B. (2003). Epidemiology of urinary tract infections: incidence, morbidity, and economic costs. Disease-a-Month, 49(2), 53-70. <https://doi.org/10.1067/mda.2003.7>.  Herthelius, B.M., Hedström, K.G., Möllby, R. et al. Pathogenesis of urinary tract infections — Amoxicillin induces genital escherichia coli colonization. Infection 16, 263–266 (1988). https://doi.org/10.1007/BF01645066  Houton, T.M. (2001). Recurrent urinary tract infection in women. International Journal of Antimicrobial Agents, 17(4), 259-268. <https://doi.org/10.1016/s0924-8579(00)00350-2>.  Nivel Primary Care Database; Nivel Zorgregistraties Eerste Lijn  Nivel, 2024. https://www.nivel.nl/nl/zorg-en-ziekte-in-cijfers/cijfers-ziekten-op-jaarbasis  Rotjanapan, P., Dosa, D. & Thomas, K.S. (2011). Potentially inappropriate treatment of urinary tract infections in two Rhode Island nursing homes. Archives of Internal Medicine, 171(5), 438-443. <https://doi.org/10.1001/archinternmed.2011.13>.  Sabih, A. & Leslie, S.W. (2023). Complicated Urinary Tract Infections. StatPearls – NCBI Bookshelf. Assessed on 09-11-2024, from Complicated Urinary Tract Infections - StatPearls - NCBI Bookshelf.  Smith, James P. Hughes, Thomas M. Hooton, Pacita Roberts, Delia Scholes, Andy Stergachis, Ann Stapleton, Walter E. Stamm, Antecedent Antimicrobial Use Increases the Risk of Uncomplicated Cystitis in Young Women, Clinical Infectious Diseases, Volume 25, Issue 1, July 1997, Pages 63–68, <https://doi.org/10.1086/514502>  Stapleton A. E., The Vaginal Microbiota and Urinary Tract Infection. Microbiology Spectrum 2016 Vol. 4 Issue 6 Pages 10.1128/microbiolspec.uti-0025-2016  VanderWeele T.J., Principles of confounder selection. Eur J Epidemiol 2019 Vol. 34 Issue 3 Pages 211-219  Worby C. J., Olson B. S., Dodson K. W., Earl A. M. and Hultgren S. J.. Establishing the role of the gut microbiota in susceptibility to recurrent urinary tract infections. The Journal of Clinical Investigation 2022 Vol. 132 Issue 5. |

**Appendix A:**Patient Brochure and Poster ELAN Datawarehouse (version April 2019)


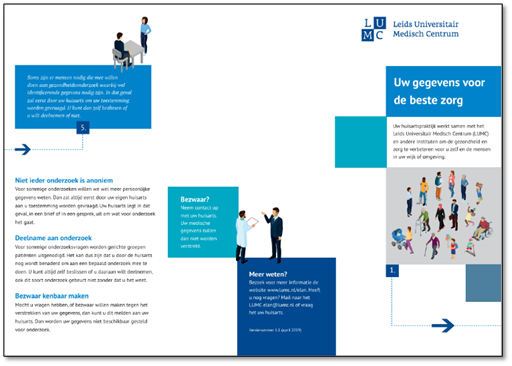


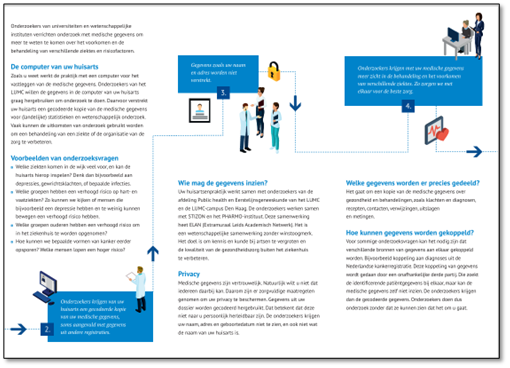

Supplement: Research Protocol [file mmc2.docx]
